# Characterising the Canine Oral Microbiome by Direct Sequencing of Reverse-Transcribed rRNA Molecules

**DOI:** 10.1371/journal.pone.0157046

**Published:** 2016-06-08

**Authors:** James E. McDonald, Niels Larsen, Andrea Pennington, John Connolly, Corrin Wallis, David J. Rooks, Neil Hall, Alan J. McCarthy, Heather E. Allison

**Affiliations:** 1 School of Biological Sciences, Bangor University, Deiniol Road, Bangor, Gwynedd, LL57 2UW, United Kingdom; 2 Danish Genome Institute, Skt. Lucas Kirkeplads 8, 8000, Aarhus C, Denmark; 3 Institute of Integrative Biology, University of Liverpool, Crown Street, Liverpool, L69 7ZB, United Kingdom; 4 WALTHAM^®^ Centre for Pet Nutrition, Freeby Lane, Waltham-on-the-Wolds, Melton Mowbray, LE14 4RT, United Kingdom; University of Minnesota, UNITED STATES

## Abstract

PCR amplification and sequencing of phylogenetic markers, primarily Small Sub-Unit ribosomal RNA (SSU rRNA) genes, has been the paradigm for defining the taxonomic composition of microbiomes. However, ‘universal’ SSU rRNA gene PCR primer sets are likely to miss much of the diversity therein. We sequenced a library comprising purified and reverse-transcribed SSU rRNA (RT-SSU rRNA) molecules from the canine oral microbiome and compared it to a general bacterial 16S rRNA gene PCR amplicon library generated from the same biological sample. In addition, we have developed BIONmeta, a novel, open-source, computer package for the processing and taxonomic classification of the randomly fragmented RT-SSU rRNA reads produced. Direct RT-SSU rRNA sequencing revealed that 16S rRNA molecules belonging to the bacterial phyla *Actinobacteria*, *Bacteroidetes*, *Firmicutes*, *Proteobacteria* and *Spirochaetes*, were most abundant in the canine oral microbiome (92.5% of total bacterial SSU rRNA). The direct rRNA sequencing approach detected greater taxonomic diversity (1 additional phylum, 2 classes, 1 order, 10 families and 61 genera) when compared with general bacterial 16S rRNA amplicons from the same sample, simultaneously provided SSU rRNA gene inventories of Bacteria, Archaea and Eukarya, and detected significant numbers of sequences not recognised by ‘universal’ primer sets. *Proteobacteria* and *Spirochaetes* were found to be under-represented by PCR-based analysis of the microbiome, and this was due to primer mismatches and taxon-specific variations in amplification efficiency, validated by qPCR analysis of 16S rRNA amplicons from a mock community. This demonstrated the veracity of direct RT-SSU rRNA sequencing for molecular microbial ecology.

## Introduction

Microbial communities comprise diverse consortia of Bacteria, Archaea and Eukarya, such that elucidating their true composition and diversity is often impossible. The use of ribosomal RNA gene sequences as phylogenetic markers revolutionised the study of molecular evolution, phylogeny and ecology in all living organisms [1–3]. Consequently, our appreciation of microbial diversity has benefited enormously from SSU rRNA gene analyses based on the 16S ribosomal RNA gene of Bacteria and Archaea and the 18S ribosomal RNA gene of Eukarya, providing a phylogenetic framework for the classification and assessment of microbial diversity in any given environment without the requirement for isolation and cultivation [4]. Contemporary studies often rely on 16S rRNA gene profiling via PCR amplification with general SSU rRNA gene primer sets to provide an overview of the taxonomic composition of a microbial community or microbiome [e.g. [5,6]]. This approach has transformed our understanding of global microbial diversity, and exponentially increased the number of ‘uncultivated’ representatives of the universal phylogenetic tree [7].

There are however well-established limitations to PCR-based approaches for microbial community analysis, and the biases have two major sources: 1) different genomic DNA templates vary in PCR amplification efficiencies, affecting both detection of taxa and estimates of their relative abundance [8–13]; 2) PCR primer sets can only be designed using available sequences in public repositories, and consequently, may not be inclusive of some novel or divergent taxa that have not previously been described. Furthermore, the introduction of relaxed specificity and degeneracy in primer design provides only a very limited expansion of this. It has been estimated that certain ‘universal’ PCR primer sets miss half of the microbial rRNA gene diversity [14,15]. Consequently, rRNA gene inventories derived from PCR amplicons miss a proportion of unexplored diversity and provide potentially misleading estimates of abundance, especially if the unidentified taxa are present in significant numbers. Furthermore, most molecular microbial ecology studies focus on microorganisms belonging to only one of the three domains; usually bacterial 16S rRNA genes.

Previous studies have used direct ‘total RNA metatranscriptome’ sequencing to avoid the biases associated with PCR-based rRNA gene diversity determinations [16,17]. Analysis of SSU rRNA sequences obtained via the ‘Double RNA approach’ (sequencing of the total metatranscriptome; mRNA and rRNA) demonstrated several taxa for which significantly different sequence counts were observed between PCR amplicon and total cDNA sequenced from the same sample [16,18]. Direct rRNA sequencing is therefore a promising approach for microbiome analysis, obviating biases associated with PCR, enabling simultaneous analysis of bacterial, archaeal and eukaryotic diversity and providing information on the number of rRNA molecules of component taxa.

However, a limitation of the total metatranscriptome sequencing approach is the high proportion of non-SSU rRNA sequence reads obtained (55 to 82%, [16–19]). Li and colleagues [18] recently described an approach for the gel-purification, reverse-transcription (with random primers) and shotgun sequencing of rRNA from activated sludge and anaerobic sludge samples, and demonstrated that comparative RT-PCR with general bacterial 16S rRNA gene primers under-represented four taxa. By comparing the diversity of shotgun rRNA reads with general bacterial PCR amplicons generated from the same cDNA sample across a homologous region of the 16S rRNA gene (variable region 3, V3), the diversity index observed for shotgun rRNA-derived sequences was greater than that obtained via PCR-based analysis of the corresponding sample [18]. However, approximately only a third (32–36%) of the RT-SSU rRNA sequences covered the V3 region, meaning that only a small proportion of the reads could be processed and compared with PCR-based diversity from the same gene region. Despite the promise of RT-SSU rRNA sequencing, further improvements in both the methodology for SSU rRNA sequencing and analysis of SSU rRNA datasets are required, including experimental validation of the observed differences between RT-SSU rRNA and 16S rRNA gene amplicon based datasets from the same sample.

The RT-SSU rRNA sequencing strategy exploits the fact that ribosomal RNA is very abundant in the cell. Although variations in the number of rRNA gene copies in the genome and the number of SSU rRNA molecules transcribed (a proxy for metabolic activity) for each species being studied will undoubtedly affect the read density of species detected, we believe that direct RT-SSU rRNA sequencing has merit for inferring estimates of relative species abundance *in situ* that can be further validated by independent techniques. This is because, unlike DNA-based PCR approaches, this technique will specifically detect the rRNA molecules of species within the microbiome. Despite the fact that the detection of rRNA molecules does not always correspond to microbial growth rate and activity, and that rRNA can be retained in dormant cells [20], direct sequencing of rRNA molecules has the advantage of avoiding PCR-associated biases, primer mismatches and by definition, is more likely to identify ‘active’ species of importance within the microbiome.

We hypothesised that the direct sequencing of reverse-transcribed rRNA molecules from the canine oral microbiome obviates the biases associated with PCR-based 16S rRNA gene analysis, and provides more complete descriptions of microbial diversity in the canine oral cavity, a complex and diverse host-associated community predicted to comprise a milieu of Bacteria, Archaea, fungi and protozoa [21]. This study had three aims: 1) to compare direct RT-SSU rRNA sequencing with PCR-based analysis of microbial diversity in the canine oral cavity; 2) to independently validate observed discrepancies in taxon abundance between 16S rRNA gene PCR amplicon and RT-SSU rRNA datasets by qPCR analysis of mock communities, and specific identification of primer mismatches, and 3) to develop a bioinformatics pipeline (BIONmeta) for the rapid and accurate classification of RT-SSU rRNA sequence datasets. We demonstrate that RT-SSU rRNA sequencing coupled with analysis using the BIONmeta software package provides an unbiased and simultaneous assessment of the diversity of Bacteria, Archaea and Eukarya from the same sample, with the potential for detecting new centres of variation that are currently missed by ‘universal’ primer sets.

## Materials and Methods

### Collection of canine plaque samples

Supra-gingival plaque was collected from ten Labrador retrievers and ten miniature Schnauzers selected from a group of dogs undergoing weekly plaque collections. Plaque samples were collected from dogs housed at the WALTHAM Centre for Pet Nutrition, who owned the dogs and gave permission for their animals to be used in this study, and the studies were approved by the WALTHAM Animal Welfare and Ethical Review Body. None of the dogs received tooth brushing and all were fed a variety of diets. Plaque samples were either collected prior to feeding or at least one hour after feeding. Supragingival plaque was collected from teeth of individual dogs by scraping plastic loops (Appleton Woods, UK) along the tooth surface. The plaque from each dog was placed into separate cryovials containing Ringers Solution (Oxoid). The samples were snap frozen in liquid nitrogen and stored at -80°C.

### Nucleic acid extraction from canine plaque

DNA and RNA was co-extracted from individual canine plaque samples (n = 20) according to the hexadecyltrimethylammonium bromide (CTAB) and phenol/chloroform/isoamyl alcohol (25:24:1) extraction protocol of Griffiths *et al*. [22] and stored at -80°C in nuclease free water. Due to the low yields of nucleic acids retrieved from canine plaque samples, and to ensure that a sufficient quantity of SSU rRNA molecules was retrieved for reverse transcription (2 μg), it was not possible to pool the plaque samples in equimolar amounts as this would have resulted in an insufficient yield of SSU rRNA. Consequently, the entire quantity of nucleic acid extracted from each individual canine plaque sample was combined into four pools of five samples prior to gel extraction and purification of genomic DNA and Small-SubUnit rRNA.

### Gel extraction and purification of genomic DNA and Small-SubUnit rRNA

Nucleic acids extracted from canine plaque samples were pooled and visualised in 1% low melting point agarose (Sigma-Aldrich) gels following electrophoresis. Nucleic acids corresponding to genomic DNA (≥ 20 Kb) and Small-SubUnit rRNA (16S and 18S, ca. 1 Kb) were excised from the agarose gel for purification.

Genomic DNA was purified from the agarose gel slice using the QiaQuick Gel Extraction kit (Qiagen) following the manufacturer’s protocol, and purified DNA was eluted into nuclease-free water and stored at -20°C until required. SSU rRNA was purified from agarose gels using β-Agarase I (New England Biolabs) following the manufacturer’s protocol with two modifications: 30 units of RNasin Plus Ribonuclease inhibitor (Promega) and 3 units of Turbo DNA-free (Ambion) were added. SSU rRNA was subsequently purified by precipitation with ¼ volume 10 M ammonium acetate and 2 x vol. 100% ice-cold ethanol and incubated at -80°C for 30 min. Following centrifugation at 18,000 g for 15 min, the RNA pellet was washed in 70% ethanol, resuspended in nuclease-free water and stored at -80°C until required.

Following purification, SSU rRNA and DNA from the four pools of five canine plaque samples were combined into a final pool of 20 samples prior to reverse transcription or 16S rRNA gene PCR, respectively.

### Reverse-transcription of SSU rRNA into double-stranded cDNA

Two micrograms of gel extracted and purified SSU rRNA from the pooled canine plaque samples was reverse-transcribed using a Just cDNA™ Double-Stranded cDNA Synthesis Kit (Agilent Technologies) following the manufacturer’s protocol and using random primers (9 mers, Agilent Technologies). Double-stranded cDNA was stored at -20°C prior to library preparation for 454 pyrosequencing (Accession no: SRR830919).

### 16S rRNA gene PCR amplification of canine plaque DNA

PCR reactions were performed in 50 μl volumes containing: 0.2 mM each primer V1-63f 5’-GCCTAACACATGCAAGTC-3' [23] and 518r 5’-ATTACCGCGGCTGCTGG-3' [24], 0.2 mM each dNTP, 1 x Phusion HF buffer (Finnzymes), 0.5 units Phusion™ High-Fidelity DNA Polymerase (Finnzymes), 10 ng of pooled canine plaque DNA and ddH_2_O. PCR cycling conditions were as follows; 98°C for 45 s, 20 cycles of 98°C for 10 s, 55°C for 30 s and 72°C for 15 s, and a final extension of 72°C for 8 min. To minimise PCR bias, 20 cycles of amplification were performed in 8 separate replicate assays, and the PCR reactions were subsequently pooled. PCR amplification products were visualised using 1% agarose gel electrophoresis and fragments of the expected size (~460 bp) were excised from the agarose gel and purified using a QiaQuick Gel Extraction kit (Qiagen) following the manufacturer’s protocol. Gel extracted and purified V1-V3 16S rRNA gene amplification products were subsequently pooled and quantified using a Qubit™ fluorimeter (Invitrogen) and stored at -20°C prior to library preparation for 454 pyrosequencing (Accession no: SRR830918).

### Library preparation and 454 Pyrosequencing

Fragment libraries for the GS FLX Titanium series were prepared using the PCR amplicons (Accession no: SRR830918) and RT-SSU rRNA (Accession no: SRR830919) according to the rapid library preparation method (Roche) and each library was sequenced on ¼ slide of a GS FLX plate.

### Analysis of 454 pyrosequencing data

PCR amplicon and SSU RT-RNA query sequences were quality checked and classified against the RDP [25], Greengenes [26] and Silva [27] databases using BION-meta (for description, please refer to S1–S3 methods), Qiime [28] and RDP classifier [29].

### Qiime

The MacQiime software package (version 1.8.0–20140103) was used to analyse the sequences from the PCR dataset. Briefly, all sequences were de-multiplexed and quality filtered, and reads with a minimum identity of 97% were clustered into operational taxonomic units (OTU’s). The most abundant sequences were chosen to represent each OTU, and taxonomy was assigned with the Ribosomal Database Project (RDP) classifier [29], and SILVA [27], with a minimum confidence threshold of 80%.

#### RDP classifier

Sequences from the PCR and SSU RT-RNA datasets were classified and compared using the ‘classifier’ function of the RDP pipeline [29] and the default settings.

### BION-meta

BION-meta is a new software package that, like Qiime and Mothur [28, 30], can create taxonomic overviews from raw sequence data, but with its own methods (S1–S3 Methods). Briefly, BION-meta cleans and de-replicates sequence reads, detects chimeras in PCR amplicon datasets, calculates similarities and projects these onto the taxonomy of a reference database. BION-meta handles low quality sequences well (ignores low quality regions without discarding the entire sequence read), can detect sequences with low similarity scores, can often differentiate species, works with non-amplicon data, installs from sources with a single line and is fast.

### Access to sequence data, BION-meta and the analysed dataset

These sequence data have been submitted to the NCBI Short Read Archive; PCR amplicon accession no: SRR830918, RT-SSU rRNA accession no: SRR830919. The software and its associated descriptions are available from: https://github.com/nielsl/mcdonald-et-al

### Detection of primer mismatches in RT-SSU-rRNA sequences

Query sequences derived from the RT-SSU rRNA dataset were aligned against their best-matching database sequences and used to identify RT-SSU-rRNA reads that contained one or both of the ‘universal’ 16S rRNA gene primer binding sites. Subsequently, the number of mismatches, insertions and deletions were determined for RT-SSU rRNA query sequence alignments that included the forward and/or reverse primer sites of the universal bacterial primer sets used to create the PCR amplicon library. These values were mapped to a taxonomy overview and used to determine the ratio of total primer site mismatches, insertions and deletions detected within that taxon to the number of sequences within the taxon that possessed mismatches and insertions and/or deletions (indels) in the primer binding site.

### qPCR analysis of ‘artificial’ microbial communities after PCR amplification of the16S rRNA gene

An ‘artificial’ microbial community was generated and comprised a mixture of five cloned 16S rRNA gene sequences that represented members of four different phyla and five different genera detected in the canine oral cavity (*Treponema* C10, *Cardiobacterium* E3, *Fusobacterium* E9, *Actinomyces* A9 and *Desulfomicrobium* F10), but with observed differences between their read density in the PCR and RT-SSU rRNA datasets. Plasmid DNA was extracted using a QIAprep Spin Midiprep kit (Qiagen, West Sussex), quantified using a Qubit fluorimeter, and linearised using *Hin*d III, which cuts the plasmid in one location and does not cut the 16S rRNA gene insert. Linearised plasmids were purified using a QIAquick PCR purification kit (Qiagen), quantified using a Qubit fluorimeter, and the copy number for each plasmid preparation was subsequently determined. Purity of the DNA was assessed using a Nanodrop spectrophotometer.

Prior to PCR, linearised plasmid DNA derived from each of the five 16S rRNA gene clones was combined in different quantities to simulate an ‘artificial’ canine oral microbial community, so that some sequences were more abundant than others. The final ratio of the five clone mixture (A9, C10, F10, E3 and E9) was 1:3:8:2:10 respectively, as determined by qPCR. The ‘artificial’ microbial community was subjected to PCR amplification using the same V1-3 16S rRNA gene-specific primers used to generate the canine oral 16S rRNA gene PCR amplicon library in this study (63f 5’-GCCTAACACATGCAAGTC-3' [23] and 518r 5’-ATTACCGCGGCTGCTGG-3' [24]. DNA template (1μL) was added to 49 μL of mastermix comprising of 22 μL DEPC H_2_0, 0.2 mM of each of 63f forward and 518r reverse primer, and 25 μL of Biomix Red obtained from Bioline (London). PCR cycling conditions were; 94°C (4 min), 94°C (30 seconds x number of cycles), 56°C (30 seconds x number of cycles), 72°C (30 seconds x number of cycles), 72 (10 min), hold at 4°C. To test the effect of cycle number on the final ratios, 3 separate PCR experiments were performed, each with varying rounds of amplification (10, 20 and 30 cycles). Each PCR reaction was conducted in triplicate.

To quantify the abundance of each cloned 16S rRNA gene sequence in the PCR amplicon mix produced by 10, 20 and 30 cycles of PCR with general bacterial primers, genus specific primers specific to each clone were designed using sequence alignments to locate regions of variability (S3 Table). For the generation of standard curves for the absolute quantification of plasmid copy number, linearised and purified plasmids were diluted by six 10-fold serial dilutions, representing 10^8^−10^3^ 16S rRNA gene copies per qPCR assay. Each dilution in the standard curve was assayed in triplicate. Five μL of the ‘artificial’ mixed microbial community was combined with 45 μL of mastermix containing 19 μL DEPC H_2_0, 0.5μL forward primer, 0.5 μL reverse primer and 25μL Sensimix SYBR Green No ROX (x2) obtained from Bioline (London). The reaction was optimized for each clone in order to find the melting temperature (Tm), extension time and primer concentration that would give the highest efficiency percentage and an R^2^ value close to 1. For each standard curve, a non-template control (NTC) was also run alongside the serial dilutions, in order to check for non-specific amplification.

To quantify the post-PCR abundance of each 16S rRNA gene sequence, genus specific primers were used in qPCR assays in conjunction with clone-specific standard curves for the absolute quantification of gene copy number of each 16S rRNA gene sequence in the artificial microbial community. Amplicon mixtures derived from the artificial community after 10, 20 and 30 cycles of PCR were diluted to appropriate levels so that the obtained Ct values would fall within the range of the standard curves, and added to qPCR assays for quantification of each 16S rRNA gene sequence type as described above.

## Results and Discussion

### Description of the canine oral microbiome using RT-SSU rRNA sequencing

SSU rRNA relative abundances determined by the RT-SSU rRNA sequencing approach and using BION-meta and SILVA database version 115 for taxonomic classification revealed a canine oral microbiota dominated by Bacteria (99.5%) with only a small proportion of archaeal (0.01%) and eukaryotic (0.46%) SSU rRNA detected (Table 1). This is consistent with previous reports that Archaea represent only a very small fraction of the oral microbiome, with diversity restricted to a few phylotypes [21]. Here, RT-SSU rRNA sequence data are in agreement with previous studies, which suggest that oral Archaea are restricted to members of the phylum Euryarchaeota [31].

**Table 1:** Abundance of the sequenced reverse-transcribed Small Sub-Unit rRNA molecules from the canine oral cavity. Domain- and phylum level classification and abundance of Archaea, Bacteria and Eukarya using BION-meta and SILVA database version 115. Only phyla with a relative abundance > 0.1% have been included.

Domain-level read density (%)					
Archaea		Bacteria		Eukarya	
0.01		99.53		0.46	
Phylum-level read density (% contribution to domain-level abundance)		Phylum-level read density (% contribution to domain-level abundance)		Phylum-level read density (% contribution to domain-level abundance)	
*Euryarchaeota*	100	*Actinobacteria*	3.6	*Amoebozoa*	0.5
		BD1-5	0.1	*Metamonada*	87.9
		*Bacteroidetes*	26.2	*Chordata*	11.6
		Candidate division SR1	1.5		
		Candidate division TM7	0.4		
		*Chlorobi*	3.0		
		*Chloroflexi*	0.3		
		*Firmicutes*	10.8		
		*Fusobacteria*	0.5		
		*Proteobacteria*	41.6		
		*Spirochaetes*	10.3		
		*Synergistetes*	0.9		
		*Tenericutes*	0.5		
Total	100	Total	100	Total	100

Eukarya represented 0.46% of the total SSU rRNA in the canine plaque samples, and these sequences represented two protozoan phyla that have been previously detected in the oral cavity [21] and unsurprisingly, a small proportion of sequences belonging to the phlyum *Chordata* (Table 1), to which canines belong. The eukaryotic population was dominated by protozoa of the genus *Trichomonas (*phylum *Metamonada*) that represented 87.9% of the eukaryotic SSU rRNA and 0.4% of the total SSU rRNA. In addition, a singleton sequence belonging to the genus *Entamoeba* (phylum *Amoebozoa*) was also detected. Both *Trichomonas* and *Entamoeba* spp. are established as inhabitants of the oral cavity in humans [21].

Our search of bacterial SSU rRNA sequences against the SILVA [27] database revealed that sequences belonging to the bacterial phyla *Actinobacteria*, *Bacteroidetes*, *Firmicutes*, *Proteobacteria* and *Spirochaetes*, were the most abundant (92.5% of total bacterial SSU rRNA) (Table 1), and this is totally in accord with previous studies [21,32–34]. With the exception of candidate division BD1-5, the remaining phyla identified in the canine oral cavity by RT-SSU rRNA sequencing here (*Chlorobi*, *Chloroflexi*, *Fusobacteria*, Candidate division SR1, *Synergistetes*, *Tenericutes* and Candidate division TM7) were all detected in the previous 16S rRNA gene clone library based canine microbiome study of Dewhirst *et al*. [32], albeit with different read densities. However, Dewhirst *et al*. [32] also demonstrated significant PCR bias attributed to the application of ‘universal’ PCR primer pairs, because cumulatively the two ‘universal’ 16S rRNA primer pairs used to generate clone libraries failed to detect members of seven phyla (*Chloroflexi*, *Fusobacteria*, *Tenericutes* and Candidate divisions GN02, SR1, TM7 and WPS-2) that were detected using other more selective primer pairs [32]. Consequently, 16S rRNA gene clone libraries generated with four separate primer pairs (two universal primer pairs, and a *Bacteroidetes*- and *Spirochaetes*-selective primer pair) were required to detect the same major phyla identified here using our direct RT-SSU rRNA approach. The data in Table 1 therefore confirm that pyrosequencing cDNA generated by reverse transcription of fractionated 16S and 18S rRNA can simultaneously resolve the identity and relative abundance of major microbial taxa across all three domains of life in a single sample. These data are a resource for the design and optimization of more inclusive taxon-specific PCR primer sets and probes for a more detailed investigation of their taxonomy and ecology.

Li and colleagues [18] recently reported a similar method for the purification, reverse-transcription and pyrosequencing of SSU rRNA from activated sludge and anaerobic sludge, and also detected a greater microbial diversity in datasets derived from directly sequenced reverse-transcribed rRNA when compared with 16S rRNA gene amplicons. In their study, Li *et al*. [18] generated 16S rRNA gene amplicons from PCR analysis of first-strand synthesized cDNA, whereas here we compare microbial community composition from (i) RT-SSU rRNA and (ii) DNA-based 16S rRNA gene PCR amplicon datasets derived from the same canine plaque sample.

### Comparison of canine microbiome diversity via RT-SSU rRNA and 16S rRNA gene PCR amplicon sequencing

**Fig 1:**
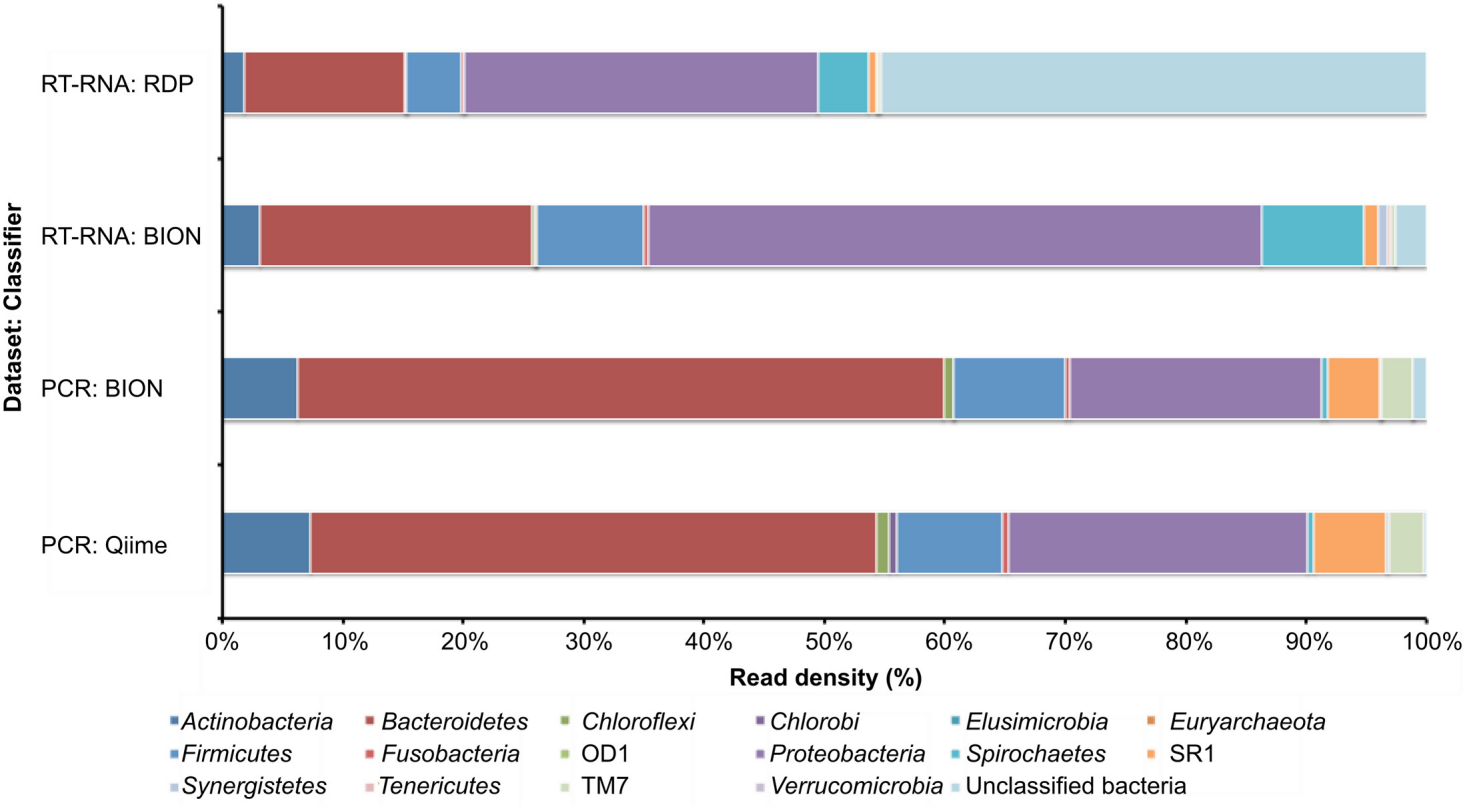
Comparison of phylum level classification of PCR amplicon and RT-SSU rRNA sequence reads derived from canine plaque samples. BION-meta was used to classify and compare sequence reads obtained from 16S rRNA gene PCR amplicons and RT-SSU rRNA from the same canine plaque sample. In order to compare the accuracy of BION-meta, the 16S rRNA amplicon dataset was also classified using Qiime and RT-SSU rRNA using the RDP classifier. The RDP database was used as a reference dataset for sequence classification with all three classifiers (RDP classifier, BION-meta and Qiime). Sequence QC and classification statistics are provided in S1 Table.

We sequenced RT-SSU rRNA molecules and general bacterial 16S rRNA gene PCR amplicons from nucleic acids extracted from the same canine plaque samples. For comparative analyses of the PCR amplicon and RT-SSU rRNA sequence output (248,760 and 257,043 sequence reads, respectively) (S1 Table), we examined the diversity and read densities of each dataset using BION-meta, and benchmarked these data against the outputs of Qiime [28] for the PCR amplicon library data, or to the RDP classifier [29] for the RT-SSU RNA dataset. BION-meta (S1 methods) is a computer package for rRNA based bacterial community analysis and was developed here to process, quality check and classify the RT-SSU rRNA sequence reads, and is a necessary development to address the bioinformatics challenge presented by the heterogeneous nature of a fragmented RT-SSU rRNA library. BION-meta provided similar classification data for both libraries compared to the widely used and validated programs Qiime [28] and RDP classifier [29], (Fig 1 and S1 and S2 Tables). Statistics for sequence numbers following processing, quality checks, chimera removal and taxonomic classification of the sequence datasets using each program are presented in S1 Table.

The amplicon library was prepared using a universal bacterial primer pair targeting a ca. 460 bp region of the 16S rRNA gene containing the variable regions 1–3 [23,24]; the DNA serving as the template was extracted simultaneously with RNA from the same plaque sample. Although several sets of universal bacterial 16S rRNA gene PCR primers are available [e.g. [10–13,21,22,31]], this primer pair was selected here for three reasons: 1) it has specificity for all cloned sequences within a general bacterial 16S rRNA gene clone library derived from the canine oral cavity [32]; 2) *in silico* comparative taxonomic classification of these cloned sequences [32] corresponding to V1-3, V5-V6 and V4 regions demonstrated that the V1-3 amplicon provided the greatest taxonomic resolution of the samples (data not shown); and 3) the primers produced the longest amplicon length compared to the other ‘universal’ primer sets.

While comparisons between the PCR amplicon and RT-SSU rRNA data derived from the same pooled plaque sample (both classified using BION-meta) revealed similar composition at the phylum level, there were distinct differences in the read density of sequences for some phyla (Fig 1). The PCR based approach indicated higher numbers of *Actinobacteria*, *Bacteriodetes*, *SR1* and *TM7* and lower numbers of *Proteobacteria* and *Spirochaetes* than RT-SSU rRNA sequencing (Fig 1). Previous studies [33,34] using PCR amplification and high-throughput sequencing to characterize the microbial composition of canine subgingival plague have identified high numbers of Gram negative bacteria associated with health, and a shift to Gram positive communities with the onset of disease [33,34]. Unlike these studies, the canine plaque samples analysed here were from supragingival plaque, which would be expected to have a slightly different composition when compared to subgingival plaque [21]. However, the PCR amplicon profiles observed here shared some similarities with the profiles described previously with respect to the relative proportions of members of the *Bacteriodetes* and *Proteobacteria* with a ratio of 26.5:16.5 [33,34], whilst this almost reverses in the RT-SSU rRNA data set (Fig 1).

**Fig 2:**
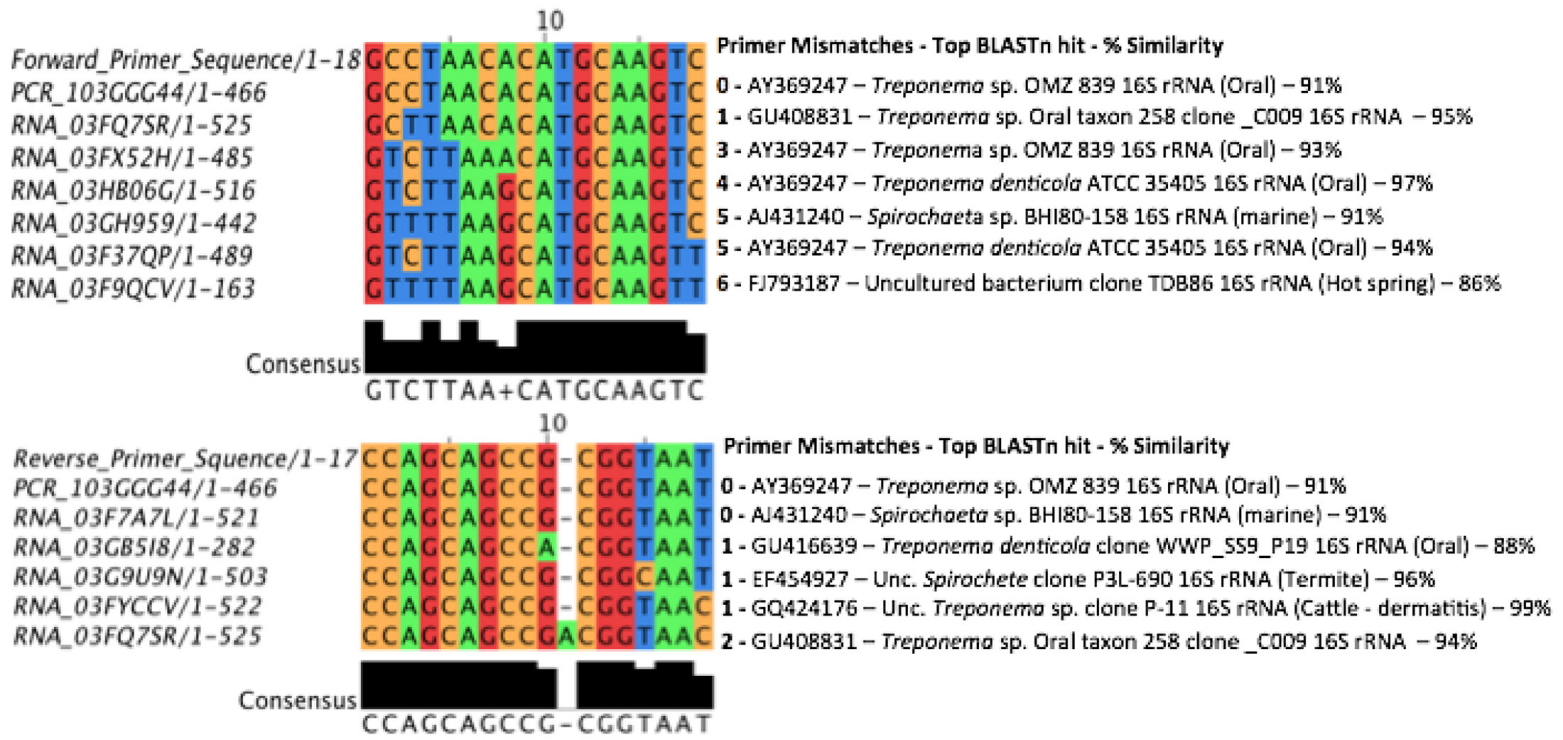
Alignment of sequence reads from the 16S rRNA gene PCR amplicon and RT-SSU rRNA datasets classified as belonging to the phylum *Spirochaetes*. Sequence reads obtained from each dataset were de-replicated using CD-HIT (http://weizhong-lab.ucsd.edu/cd-hit/) and representative sequences for each OTU group aligned against ‘good’ quality reference *Spirochaete* sequences from the Ribosomal Database Project website (http://rdp.cme.msu.edu/). Sequence names (left column) beginning with PCR are from the PCR amplicon dataset and sequences beginning with RNA are from the RT-SSU rRNA dataset. The column to the right of the alignment highlights the number of mismatches between the sequence group and the primer site, followed by the Genbank accession number of the closest BLASTn match to that group. The environmental source of the closest reference sequence is presented in parentheses where not stated in the BLAST description and the % similarity to our query sequence is also shown. The sequence of the forward and reverse primers used to create the PCR amplicon library (63f 5’-GCCTAACACATGCAAGTC-3' and the reverse complement of 518r 5’-ATTACCGCGGCTGCTGG-3') are shown as the top sequence in each alignment.

Whilst one might expect a discrepancy between the number of sequence reads obtained when comparing 16S rRNA gene copies with 16S rRNA molecule numbers for a given taxon, the considerably lower read density of spirochaete sequences obtained by the PCR-based approach, read density of 0.4% and 8.5% for PCR vs. RT-RNA, respectively, is noteworthy. Previous, general bacterial PCR amplicon inventories of the oral microbiome suggest that *Spirochaetes* represent a minor fraction of the canine [32–34] or human microbiome [21,32,35], but microscopy studies have demonstrated that between 8 and 54% of bacterial cells from human oral plaque were *Spirochaetes* [35,36]. This was also a feature of the study of Li *et al*. [18], where it was reported that *Spirochaetes* were underestimated in PCR amplicon libraries of anaerobic sludge samples. This underestimation of the spirochaetes has been attributed to PCR primer bias [32,35], and our data strongly support this assertion. To investigate this further, we aligned spirochaete sequences generated from both the PCR amplicon and SSU rRNA libraries with reference spirochaete sequences from the Ribosomal Database Project website (http://rdp.cme.msu.edu/) and identified mismatches in the RT-SSU rRNA sequences that contained the homologous binding sites for the general bacterial primers used to generate the PCR amplicon library (Fig 2). Spirochaete sequences from the RT-SSU rRNA dataset had up to six mismatches in the forward primer (63f) binding site and two mismatches with the reverse primer (518r) binding site, demonstrating that PCR amplification using this primer pair would be unlikely to detect these sequences, thus providing an explanation for the under-representation of spirochaetes using PCR-based analysis here and elsewhere [32,35].

### Detection of greater sequence diversity and potential novel taxa via RT-SSU rRNA sequencing

**Fig 3:**
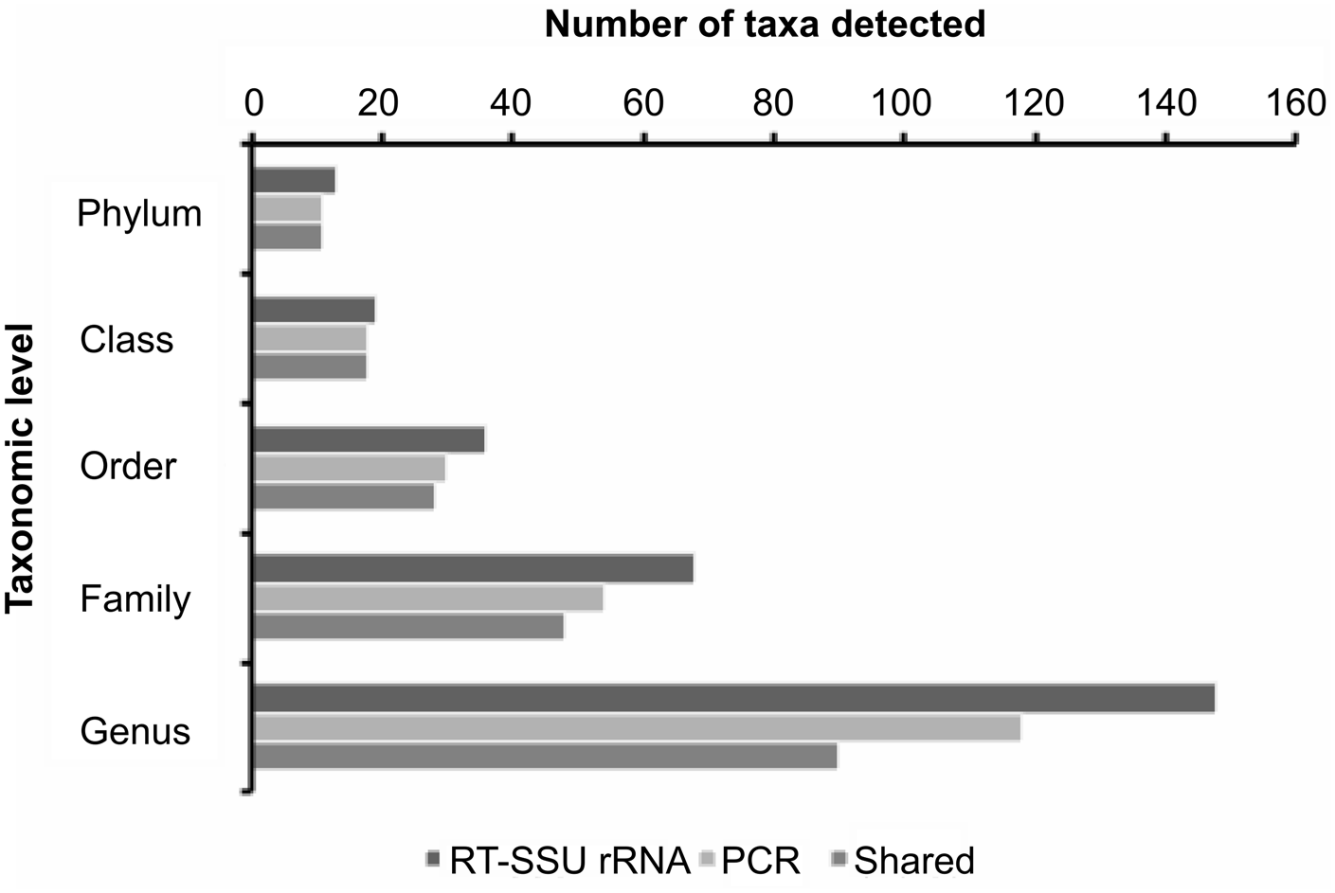
Comparison of the number of taxa detected at each phylogenetic rank in the 16S rRNA gene amplicon library (PCR) and the sequenced RT-SSU rRNA library (RT-rRNA) generated from canine plaque samples. The datasets were compared using the command line RDP library compare function. Bars denote the number of taxa detected at each phylogenetic level in the RT-SSU rRNA and 16S rRNA gene amplicon dataset, respectively, and the number of taxa that were common to both datasets.

Classification of sequence reads using the RDP classifier indicated that 45% of the RT-SSU rRNA reads represented ‘unclassified bacteria’ (Fig 1), suggesting the presence of potentially novel centres of variation that have previously evaded detection by both cultivation-based and amplicon sequencing studies. Furthermore, the differences in the number of taxa detected at each taxonomic rank (using RDP classifier) by both techniques are shown in Fig 3. The RT-SSU rRNA method consistently detected more bacterial taxa at every taxonomic level; an additional phylum, 2 classes, 1 order, 10 families and 61 genera were detected in the RT-SSU rRNA dataset when compared with 16S PCR amplicons from the same sample (Fig 3). Li *et al*. [18] also found a greater microbial diversity index when SSU rRNA was directly pyrosequenced compared with libraries prepared by 16S rRNA gene amplification of first-strand cDNA. Furthermore, many of the sequences in our RT-SSU rRNA dataset could only be resolved above the genus level, suggesting the presence of potentially novel taxa at every phylogenetic rank (S2 Table). It should be noted that due to the randomly fragmented nature of the RT-RNA reads, it is plausible that some sequences may cover conserved areas of the 16S rRNA gene and are therefore less phylogenetically informative than reads containing variable regions. However, the BION-meta programme screens sequence reads against user-defined variable regions of the 16S rRNA gene (S1–S3 Methods) to improve the phylogenetic resolution of the reads. Furthermore, Li and colleagues [18] performed *in silico* analysis of randomly fragmented 400 bp SSU rRNA sequences to simulate reverse transcription with random primers and demonstrated that 99.4% of the sequence reads were correctly classified, providing similar population structures to the original full-length sequences from which they were derived.

### Detection of mismatches with ‘universal’ primer binding sites in RT-SSU rRNA sequences

**Fig 4:**
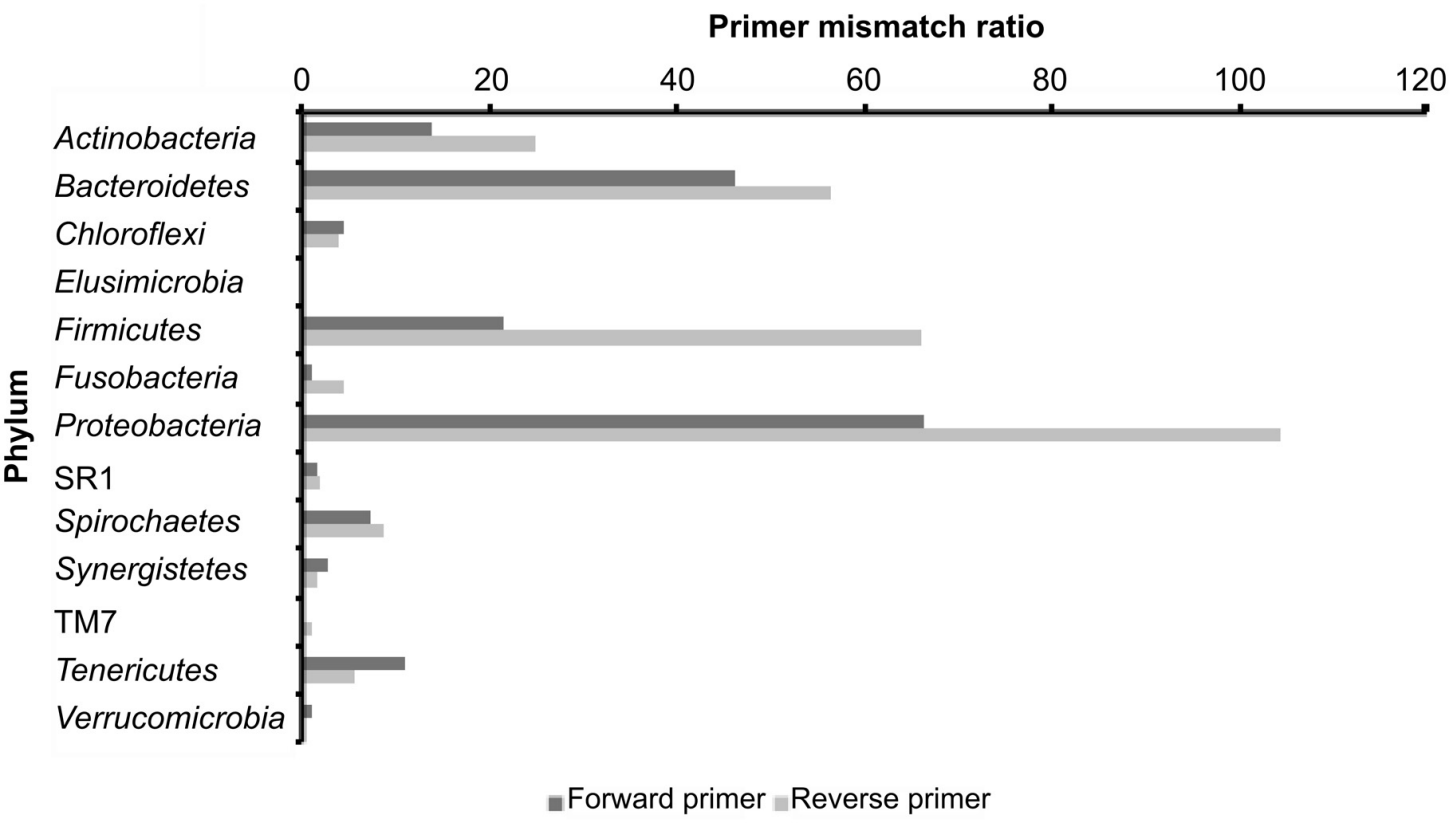
Primer mismatch ratios for phyla detected using the RT-SSU rRNA approach. RT-SSU rRNA sequences containing regions corresponding to the forward and reverse PCR primer sites used to generate the PCR amplicon library in this study were aligned against their closest database match, and the number of insertions, deletions (indels) or mutations within the primer binding site recorded. Primer mismatch ratios were calculated by dividing the total number of sequence reads containing the primer-binding site by the total number of indels and mutations recorded within the primer binding sites of those sequences.

An obvious explanation for the increased taxonomic diversity observed in the RT-SSU rRNA dataset is that PCR primers may not be inclusive of novel or divergent sequence types that have not previously been described. To investigate this, we aligned the RT-SSU rRNA query sequences with their closest database match and identified insertions, deletions and mutations present within the regions of the SSU rRNA reads that correspond to the PCR primer binding sites of the primers used to amplify the 16S rRNA gene for the amplicon library. Sequence mismatches with at least one of the primers used to generate the 16S rRNA gene amplicon library were detected in all phyla observed in the RT-SSU rRNA library, with the exception of the phylum *Elusimicrobia* (Fig 4). Previously undetected sequence diversity within the binding site of the general bacterial primer set used is therefore one explanation for the increased diversity observed in the RT-SSU rRNA dataset compared to 16S rRNA gene amplicons from the same sample. These data support our assertion that novel centres of variation are detected via the RT-SSU rRNA approach.

### Validation of PCR amplification bias in a mock community comprising taxa that are under- and over-represented in the canine oral microbiome

Primer mismatches do not explain all of the discrepancies we found for taxa that had major differences in sequence counts between the datasets. To independently demonstrate the effect of PCR amplification bias, we generated an artificial mixture of five cloned 16S rRNA genes, each possessing the universal primer binding sites used to produce our PCR amplicon library; we used canine oral bacterial taxa that were identified as under-represented (*Fusobacterium* and *Proteobacteria-Desulphomicrobium)* or over-represented (*Actinobacteria* and *Proteobacteria-Cardiobacterium*) in the PCR amplicon dataset, and one that had a similar number of sequences (*Treponema*) in the PCR and RT-SSU rRNA datasets. The members of the artificial community were mixed in known ratios of gene copy number and subjected to 10, 20 or 30 cycles of PCR.

**Fig 5:**
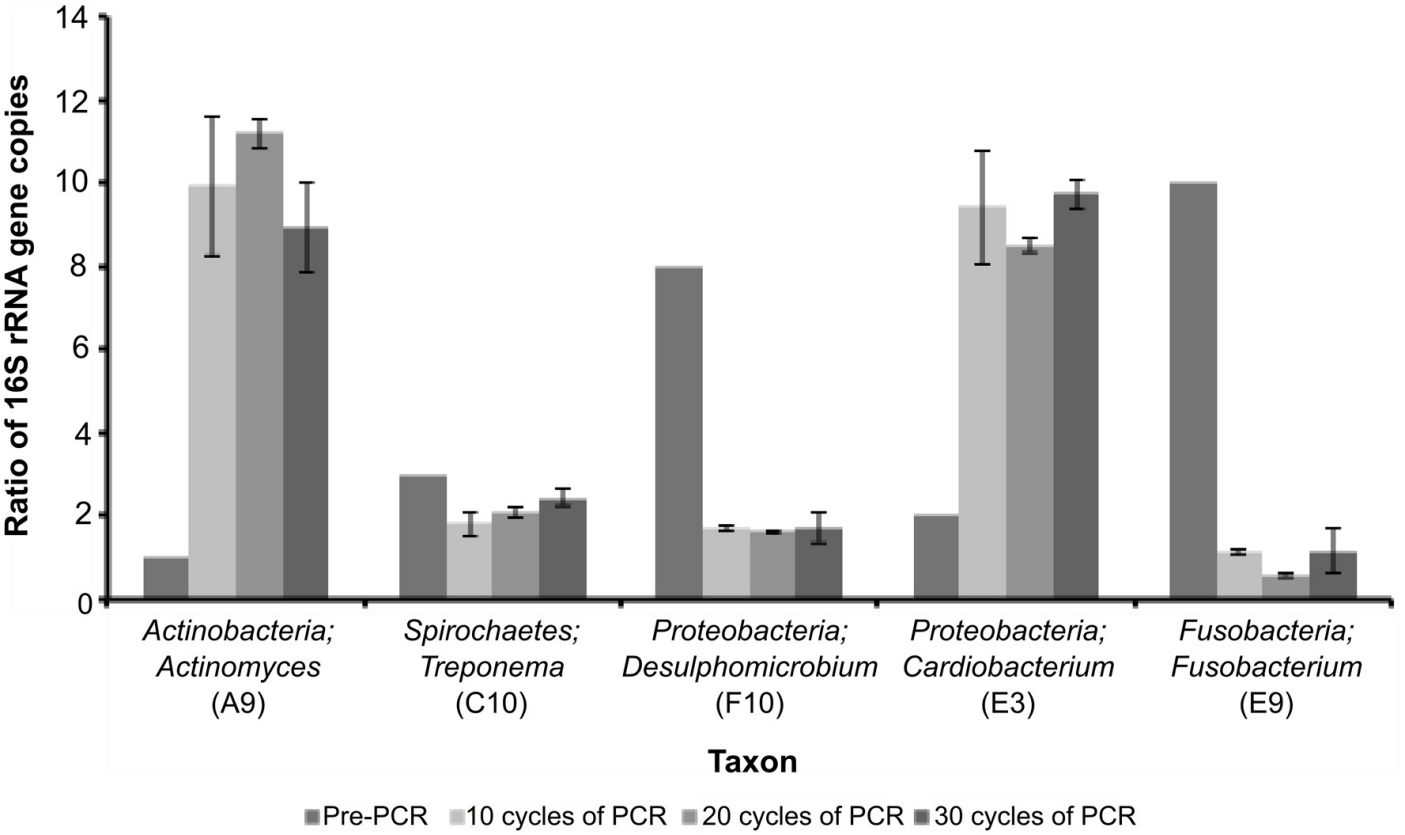
Quantitative PCR analysis of amplification efficiencies of an artificial microbial community comprising five cloned 16S rRNA genes of canine oral bacteria. The artificial community was generated by mixing ratios of known gene copy number (A9, C10, F10, E3 and E9 in the ratio of 1:3:8:2:10, respectively), followed by 10, 20 or 30 cycles of PCR amplification using the universal bacterial primer set applied in this study. The resulting community PCR amplicons were subjected to qPCR analysis using clone-specific primer sets to determine the relative ratios of each clone in the final amplification mix. Error bars represent the standard error of the mean from 3 independent biological replicates. Data from each biological replicate were obtained from three experimental replicates.

Subsequently, primer sets specific for each member of the artificial community were used to quantify the abundance of each 16S rRNA gene in the resulting amplicon pool by qPCR via a direct quantification strategy using taxon-specific standards. These data demonstrate significant differences in the amplification efficiencies of each 16S rRNA gene (Fig 5); the *Actinobacteria* 16S rRNA gene was over-represented (Pre-PCR ratio of 16S rRNA gene copies = 1, post-PCR average ratio of 16S rRNA gene copies = 10), whereas the *Fusobacterium* 16S rRNA gene was under-represented in the amplified gene pool (Pre-PCR ratio of 16S rRNA gene copies = 10, post-PCR average ratio of 16S rRNA gene copies = 1). These observations are consistent with a recent study of the canine oral microbiome in which members of various phyla are represented in varying relative abundances from the same biological sample depending on which set of ‘universal’ bacterial primer sets was used, *e*.*g*. F24+AD35/C72 [9-27F/1492-1509R], F24/Y36 [9-29F/1525-1241R] [32]. Two of the cloned 16S rRNA genes derived from separate genera of the *Proteobacteria* gave contrasting amplification efficiencies (Fig 5; *Desulphomicrobium* Pre-PCR ratio of 16S gene copies = 8, post-PCR average ratio of 16S rRNA gene copies = 2, *Cardiobacterium* Pre-PCR ratio of 16S gene copies = 2, post-PCR average ratio of 16S rRNA gene copies = 9); the spirochaete clone displayed a similar abundance to the actual ratio of gene copies in the artificial community (Fig 5).

The cloned 16S rRNA gene sequences could all be amplified by the universal bacterial primers (63f [23] and 518r [24]), and though there were a few mismatches with the 63f primer, the last 11 nucleotides at the 3’ end matched perfectly (Number of mismatches with forward primer; F10, 0; E9, A9 and E3, 1 and C10, 3). Furthermore, the comparative amplification efficiencies of each cloned template did not correlate with universal primer mismatches in the clone sequence templates. Consequently, amplification efficiencies are not controlled merely by primer recognition strength, or %GC content, but by other as yet uncharacterised properties, possibly inherent to the DNA template. Taken together, these data suggest that the failure to detect certain taxa via PCR-based approaches is a combination of several factors that include primer mismatches and differential PCR amplification efficiencies [10,12,13,37,38].

## Conclusions

Here, we describe a PCR-independent method for the characterisation of SSU rRNA genes derived from all members of the microbial community. We sequenced a library composed entirely of SSU rRNA molecules and demonstrate that by achieving this without a universal PCR amplification step, a much-extended catalogue of microbial diversity is revealed with differing population structure. Although you would expect to find differences between the population structure of a microbiome when taxonomic profiles derived from rRNA molecules and rRNA gene PCR amplicons are compared, our detection of a greater diversity and proportion of spirochaetes for which mismatches with the primers used to generate the PCR amplicon library were observed confirms that at least some of these differences are due to biases associated with PCR itself rather than differences arising from the analysis of rRNA molecules vs. rRNA gene amplicons.

There are obvious discrepancies between cellular SSU rRNA gene copy and SSU rRNA molecule numbers across taxa that must be taken into consideration when inferring taxon abundance in microbiomes, and rRNA abundance data does not represent a proxy for the actual relative abundance of cells in the sample. However, these data can, and should, be further validated by complimentary and independent approaches such as fluorescent *in situ* hybridization, qPCR validation and metagenomics. Metagenome sequencing also offers a PCR-independent taxonomic assessment of microbial communities, and combined with programmes such as EMIRGE [39] enables the assembly of rRNA gene sequences from metagenomic datasets, and may also be applied for the analysis of RT-SSU rRNA sequence reads generated using Illumina technology. However, metagenomic analyses provide genome-wide functional and phylogenetic sequence data, which by their nature, are depleted in SSU rRNA reads that represent only a tiny proportion of the genome. Furthermore, many of the shotgun metagenomic reads are taxonomically uninformative, or at best only indicative.

A limitation of the RT-SSU rRNA sequencing approach at the time of this study was the quantity of SSU rRNA required for reverse transcription and sequencing library preparation (2 μg). However, due to advances in available sequencing technologies (e.g. Illumina paired end sequencing) and library preparation kits, the quantity of input RNA required for library preparation has decreased significantly, allowing greater replication of samples and a more robust statistical comparison of the data in future studies. Consequently, technological developments in this rapidly evolving field make the application of the direct RT-SSU rRNA sequencing approach, or even sequencing of total RNA without any rRNA purification, an inexpensive and effective method for the PCR-independent analysis of microbial community composition.

Here, we demonstrate that RT-SSU rRNA sequencing of the canine oral microbiome combined with analysis using BION-meta, a new software pipeline (S4 Table), provides an indiscriminate and simultaneous determination of microbial diversity and rRNA molecule number from all three domains, within the same sample. The technique and software package described here will take molecular microbial ecology forward, identifying new taxa, and providing microbial community structure and diversity determinations that are made more meaningful by the absence of biases introduced by the application of PCR amplification and cloning.

## Supporting Information

S1 Methods Description of the BION-meta: a bioinformatics pipeline for the rapid and accurate classification of rRNA sequence datasets. (DOCX)

S2 Methods 16S PCR amplicon recipe. (DOCX)

S3 Methods RT-SSU rRNA analysis recipe. (DOCX)

S1 Table Summary table of statistics for the processing and classification of the sequence data presented in Fig 2. (DOCX)

S2 Table Classification of 16S rRNA gene PCR amplicons (Accession: SRR830918) and RT-SSU rRNA sequence reads (Accession: SRR830919) using the RDP classifier and library comparison tool. (DOCX)

S3 Table Genus-specific 16S rRNA gene PCR primer sets. Primer sets were designed for use in qPCR experiments in order to assess the change in ratio of 16S rRNA gene copies resulting from amplification of an initial artificial 5-member microbial community subjected to 10, 20 and 30 cycles of PCR with the general bacterial primer set 63f 5’-GCCTAACACATGCAAGTC-3' and 518r 5’-ATTACCGCGGCTGCTGG-3'.
(DOCX)

S4 Table Features and advantages of the BION-meta software. (DOCX)
