# Supplementary material for: Characterising the Canine Oral Microbiome by Direct Sequencing of Reverse-Transcribed rRNA Molecules
Source: PLoS One. 2016 Jun 8;11(6):e0157046. doi: 10.1371/journal.pone.0157046 (PMC4898712; doi:10.1371/journal.pone.0157046)
Supplement: S1 Methods — (DOCX) [file pone.0157046.s001.docx]

**S1 Methods. Description of the BION-meta: a bioinformatics pipeline for the rapid and accurate classification of rRNA sequence datasets.**

About BION-meta

---------------

This is an as yet unpublished open source package for microbiome analyses that takes sequence machine data as input and produces taxonomic overviews. Currently the package only analyses 16S/18S rRNA gene sequences. Development focus is on better methods, shorter run-times, more transparent results and access, and enabling inexperienced users to run and re-run flows. The aim is a package well suited for routine usage, that easily adapts to data set size and which requires little manual intervention.

The package will remain 100% open source. BION-meta was developed during 2012-13 by the Danish Genome Institute, a consulting firm. Its development was partly sponsored by the Danish Veterinary Institute and Statens Serum Institut, both in Copenhagen, Denmark. Until its own web-site is made, this temporary page has more information,

http://box.com/bion

About recipes

-------------

BION recipes consist of steps in a text file that are run in the given order; see for example the PCR.recipe link in the top panel of the PCR output. Each step has parameter keys and values that are passed on to the underlying method. Any routine or program with a command line interface can be made to appear as a recipe step.

Pipeline steps

--------------

The PCR and RT-SSU rRNA (RT) pipelines both had the following steps (see PCR.recipe and RT.recipe links in the top panel of the outputs),

1. Sequence cleaning

2. Sequence de-replication

3. Chimera filtering

4. Silva similarities

5. Silva taxonomy mapping

6. Silva profiles

7. RDP similarities

8. RDP taxonomy mapping

9. RDP profiles

10. Greengenes similarities

11. Greengenes taxonomy mapping

12. Greengenes profiles

While similar overall they differ in detail, because the two datasets are different. The method for each step is explained below. The parameter settings used with each method are explained in the respective recipes.

Total run-time was 46 and 39 minutes for the PCR and RT set, respectively, on an eight-core machine, and maximum RAM usage was around 1.5 gb. Recipes will run on more modest hardware, like laptops, but with fewer cores the duration will be proportionally longer.

Reference databases

-------------------

For the PCR data, slices were cut out of the full-length Silva and RDP alignments with just the sequences that completely span the amplicon used (in this case the V1-V3 region). The RDP alignments, which come as separate Bacterial and Archaeal versions, were joined. We could not do this with Greengenes, since the alignment routine used (NAST; Near Alignment Space Termination) alters the sequences. Using sub-datasets has three advantages: 1) lower similarities can be detected, since no match will occur in parts outside the amplicon, 2) run times are shorter, less memory is used, and 3) most importantly, there are no falsely high or low similarities between query or database partial sequences.

For the RT data, there was no amplicon or defined rRNA gene region, so relevant slices could not be obtained from the database sequences. Instead, we simply used all sequences in the databases that were at least 1250 bases long. That does not completely exclude matches between sequences with minor overlap, but requiring longer sequences resulted in too few database sequences.

We ran both datasets against two versions of Silva, the RDP and Greengenes, either including all sequences or named species only. Since this study shows differences between major groups, similar results were obtained.

For chimera checking it is also important to use sequences that span the whole amplicon, no more and no less. It is however not important to include all highly similar sequences, so the amplicon datasets were clustered, with all sequences included, to the 99% level. This gave a two- to six-fold reduction in the number of sequences.

The taxonomy in both RDP, Silva and Greengenes has various consistency issues, though Greengenes less so. For example, families and genera did not always occur at the same level in the databases. Quite elaborate routines were made to patch various issues, so that similarity scores from lower to higher levels could be summed up (see below).

Step methods

------------

1. Sequence cleaning

The cleaning steps performed, with all settings, are visible in the PCR.recipe and RT.recipe files linked to at the top of the output. Here we just summarize our cleaning methods in general terms.

Clipping. When a given sequence matches a sub-sequence or a pattern (e.g. a primer or adapter site), it is cut. The pattern can include indels, sequence motifs and secondary structure, and the cut can be made either at the end or the start of the match.

Sequence trimming. A probe sequence slides into the data sequence one step at a time but stops when a user-definable match/mismatch ratio is met (default 80%). The matching sequence is then cut. This step is typically used for trimming off primer remnants. A few bases from the real data may also disappear, but sequences are usually clustered, and then they will re-appear in the resulting consenses.

Quality trimming. A sliding window of user-definable length counts the number of bases with a specified quality percentage. If the quality requirement is satisfied within the window, then it stops and the preceding sequence disappears. Finally the bases are trimmed one by one for less than required quality.

Filtering. This does not alter sequences but keeps only those in a list that meet the given constraints. Filtering constraints can include

* Sequence match / non-match

* Pattern match / non-match

* Minimum and/or maximum quality and strictness for all bases combined

* Length, minimum and/or maximum

* GC content, minimum and/or maximum percentage

2. Sequence de-replication.

This is another rather standard step that simply converts multiple identical sequences into one. The Kyoto Cabinet key/value store was used to keep dereplicated sequences on file, which gives a good balance between speed and RAM usage.

3. Chimera filtering.

This method measures chimera-potential of single-gene DNA sequences against a well-formed reference dataset without alignment.

Inputs are one or more files with unaligned sequences and a reference dataset file (like 16S), also unaligned. Outputs are score tables plus chimera- and non-chimera sequence files along with statistics files. Currently, only two-fragment chimeras are detected, but triple-fragment chimeras usually also receive high scores.

The method works like this. A hypothetical breakpoint is moved along the query sequence, so there is a left fragment (L) and a right fragment (R) as it moves. At each position the mismatches between L and the most similar database sequence are counted and remembered; same for R. If there is a chimera, then L-mismatches should start to rise downstream from the true break-point, and the R-mismatches should rise from that point and upstream. When the amounts of these mismatches are shown for each query sequence position as a cumulative histogram, then ideally a "valley" will form, in which the bottom is the breakpoint. If the database similarities for L and R are very high, then the histogram will have low values and vice versa. Independent of similarity, the valley is sometimes deep or shallow, which reflects how different the two fragments are. This depth is then compared with how a non-chimeric version would appear, and scored by how much the difference is. This method catches all obviously false combinations of fragments from distantly related species, but also closely related ones. The most difficult task is to detect combinations where both fragments have low similarity against the database, but even here the method gives an identifiable separation between false and real chimeras. To obtain good results, the reference dataset must be well formed and contain only sequences that span the amplicon, preferably no more.

The method has not yet been systematically evaluated against existing methods. However we have run datasets through Uchime (http://drive5.com/usearch/manual/uchime_algo.html), currently the most widely used program. We found good agreement between the two and using default parameters no obvious chimeras were missed by either.

The implementation uses slightly less RAM than Uchime and is 20-25% faster, using all available CPU cores by default. This new program is by far the fastest open-source implementation, and it is probably at least as sensitive as the existing programs.

4. Silva similarities

The program "simrank2" was written to create oligo-based similarities between unaligned sequences in a query file and a reference dataset, also with unaligned sequences. It handles quality, detects low similarities and uses no index file. It is as yet unpublished.

For a given query sequence (A) and reference sequence (B), their similarity is calculated in this way: first A and B are converted to two lists of unique "words" ("k-mers") that are short sub-sequences of a fixed length in the 6-12-mer range, where 8 (the default) was used in this study. The similarity is then simply the number of common words divided by the smallest set of words in either. This ratio is then multiplied by 100 to become a percentage.

[NOTE: this oligo-percentage is very different from normal sequence similarity based on mismatches within single positions, and there is no straight relationship between the two: if word-length is 8 and every 8th base position is a mismatch, the oligo-percent is then zero, but the similarity percent is 87.5. In practice, however, the conservation patterns do not vary much between reference sequences, and the method is robust, pulling out the best reference matches.].

Simrank2 was written for three reasons,

i. No available program can simply ignore only the low quality spots in a sequence; they all operate on the entire sequence, disregarding available quality information. Often there are low quality spots in otherwise good sequences, especially in 454 pyrosequencing data, which is prone to errors, leading to a false over-estimation of diversity. Simrank2 handles quality by simply skipping over such spots: e.g. if three neighboring bases are poor, then the 10 8-mers are ignored across that overlap with these three positions. The minimum quality to use is defined in the recipe; there is a parameter for skipping non-canonical bases also and quality values are present in the reference datasets (if they are not), then they are treated similarly.

ii. No similarity program can return the best range (user definable) of similarities, however low they are. In BLAST for example, the only option is to set the similarity threshold low or high: if set to low, huge amounts of hits are returned; if set to high, low-scoring sequences are missed. In simrank2, the default is to get the top one percent of the similarities, whether they are high or low.

iii. Speed. With a word size of 8 and step length of 4, a dataset of 250,000 454 reads will be analysed on a single CPU-core in 40-70 minutes against a dataset of 1.5 million 500 bp-long reference sequences. On an 8-core machine, run time will be around 10 minutes. Run-time is roughly linearly proportional to the query sequence volume and the reference sequence volume (i.e. the number of bases). Run-time is inversely proportional to step length, whereas longer words only have a moderate speed advantage. RAM usage is typically between 500 MB and 2 GB. These parameters are fully controllable by user settings and depend on the data. This performance is much better than any other alternative, and certainly better than simrank1^*^^[[1]](#footnote-1)^*.

The output from this step is a table with a line for each query sequence that lists the best reference identifications and oligo-percent values.

5. Silva taxonomy mapping

We did not use the RDP classifier, or its logic, with BION-meta but wrote our own taxonomy mapping routines. Under our scheme, similarities for a given query sequence are simply "projected" onto the taxonomy, which is represented as a tree. The list of similarities (for the same query sequence) is like the ornaments on a Christmas tree, except they may cover multiple branches. More specifically, the mapping is performed via these steps for each query sequence, while keeping track of original read counts,

5a. The lowest level taxonomic tree node that exactly spans all the similarities is found.

5b. From the node identified in 5a, which contains all nodes below with similarities, a subtree is constructed.

5c. Nodes that have two or more leaves, but no sub-nodes, are pruned while giving the sum of the clipped read counts to the parent. The result is a truncated tree, which most often is a single node. However, when multiple taxa score equally, the read count is divided among them.

This method of painting similarities on a tree may seem crude, as there is no measure of reliability or robustness and there is no notion of "placement" of each sequence. However, three more features are being added that should address this, plus improve accuracy much beyond what other current packages can do. This simple approach can already often separate reads at the species level - and sometimes sequence level. Also, for this study, only major groups were compared for which this unrefined method is more than adequate. Indeed, as our results show, its outputs at the phylum/class level are very similar to those of the RDP classifier.

6. Silva profiles

The previous step created a binary profile with all floating point numbers in native format with no loss of information. This step is merely a formatting step, in which readable tables are created. There are four kinds of tables, each of which has a spreadsheet-ready text version as well as an HTML version.

6a. The profiles scores, untransformed. A minimum read-score can be set, so only rows with at least that minimum in some column are included.

6b. The normalized scores. Values in each column are scaled so their totals add up to a given value. That value is by default 100,000 so that the numbers are percentages times 1000.

6c. The summed scores. All taxonomic parent groups are included, each with accumulated scores for all taxa below.

6d. The summed normalized scores. As for 6b, but with the scaled values.

7-12. RDP and Greengenes

Same steps as described above for Silva, but applied to RDP and Greengenes. See

also comments in the recipes.

1. * DeSantis TZ*, et al.* (2011) Simrank: rapid and sensitive general-purpose k-mer search tool. (Translated from eng) *BMC Ecol.* 11:11 (in eng). [↑](#footnote-ref-1)
