# Supplementary material for: Characterising the Canine Oral Microbiome by Direct Sequencing of Reverse-Transcribed rRNA Molecules
Source: PLoS One. 2016 Jun 8;11(6):e0157046. doi: 10.1371/journal.pone.0157046 (PMC4898712; doi:10.1371/journal.pone.0157046)
Supplement: S2 Methods — (DOCX) [file pone.0157046.s002.docx]

**S2 Methods. 16S PCR amplicon recipe**

# -*- html -*-

# The recipe below, which is input to the run_recipe command, consists of

# steps that are run in the order given. Each step has parameter keys and

# values that are passed on to the underlying method. Any routine or program

# with a command line interface can be made to appear as a recipe step.

<recipe>

title = Domain 1-3 PCR vs Silva, RDP and Greengenes

author = Niels Larsen, James McDonald

email = niels@genomics.dk

site = Danish Genome Institute, Bangor University

# ------------------------------------------------------------------------

# SFF FORMAT CONVERSION

# ------------------------------------------------------------------------

# Not used, but works. Can convert .sff files to fastq for example, which

# BION uses as default exchange format between steps.

# <sequence-conversion>

# title = Conversion to fastq

# </sequence-conversion>

# ------------------------------------------------------------------------

# CLEANING

# ------------------------------------------------------------------------

<sequence-cleaning>

title = Sequence cleaning

quality-type = Sanger

# Clip adapter. Sub-sequence at the start were removed up to the end

# of the match. The [1,1,1] means up to one mismatch, deletion and

# insertion in the pattern sequence respectively. Only the first 20

# positions were matched against.

<sequence-clip-pattern-start>

title = Start adapter clip

pattern-string = ^ TCAGTACTGAGCTA[1,1,1]

pattern-orient = forward

include-match = no

search-distance = 20

</sequence-clip-pattern-start>

# 5' primer removal, sequence start. Clip it by deleting all positions

# before the end of the primer pattern match below. Only the first 30

# positions were considered.

<sequence-clip-pattern-start>

title = 5 primer start

pattern-string = GCCTAACACATGCAAGTC[1,1,1]

pattern-orient = forward

include-match = no

search-distance = 30

</sequence-clip-pattern-start>

# 5' primer removal, sequence end. The amplicon was shorter than the

# longest read, so those reads may have a complemented copy of the

# reverse primer. This step removes those, and all sequence that

# follows, by matching the pattern below against the last 350

# positions.

<sequence-clip-pattern-end>

title = 3 primer reverse end

pattern-string = CCAGCAGCCGCGGTAAT[1,1,1]

pattern-orient = forward

include-match = no

search-distance = 350

</sequence-clip-pattern-end>

# 3' primer removal, sequence start. Clip it by deleting all positions

# before the end of the primer pattern match below. Only the first 30

# positions were considered.

<sequence-clip-pattern-start>

title = 3 primer start

pattern-string = ATTACCGCGGCTGCTGG[1,1,1]

pattern-orient = forward

include-match = no

search-distance = 30

</sequence-clip-pattern-start>

# 3' primer removal, sequence end. The amplicon was shorter than the

# longest read, so those reads may have a complemented copy of the

# reverse primer. This step removes those, and all sequence that

# follows, by matching the pattern below against the last 350

# positions.

<sequence-clip-pattern-end>

title = 5 primer reverse end

pattern-string = GACTTGCATGTGTTAGGC[1,1,1]

pattern-orient = forward

include-match = no

search-distance = 350

</sequence-clip-pattern-end>

# Adapter remnant trimming. We found adapters, and parts of them,

# present at both ends, and frequently repeated two or three times

# upstream. These four steps (see PCR.recipe) represent our best

# effort to remove these.

<sequence-clip-pattern-end>

title = Reverse adapter at end

pattern-string = CTGAGACTGCCAAGGCACACAGGGGATAGG[2,1,1]

pattern-orient = forward

include-match = no

search-distance = 50

</sequence-clip-pattern-end>

<sequence-clip-pattern-end>

title = Reverse adapter fragment

pattern-string = AAGGCACACAGGGGAT[1,1,1]

pattern-orient = forward

include-match = no

search-distance = 50

</sequence-clip-pattern-end>

<sequence-clip-pattern-end>

title = Reverse adapter fragment

pattern-string = TGAGACTGCCAAAGGCACACA[1,1,1]

pattern-orient = forward

include-match = no

search-distance = 50

</sequence-clip-pattern-end>

# Sequence trimming, sequence end. Starting at 50 positions from

# the end, the similarity between the adapter sequence and the query

# is measured. If it is 80% or better, then the window stops

# and the sequence is cut where the match starts. If not 80% or

# better, the sequence slides towards the end and past it, so there

# is less and less overlap. The query is cut at the first match of

# 80% or better. Most often this causes no bases to be cut, or just

# the last one (and the last one is dispensable, as following

# clustering steps usually will recover it).

<sequence-trim-end>

title = Reverse adapter end trim

sequence = CTGAGACTGCCAAGGCACACAGGGGATAGG

search-distance = 50

minimum-length = 1

minimum-strict = 80%

</sequence-trim-end>

# Quality trimming, sequence start. A sliding window of length 15

# counts the number of bases with a quality of at least 97%. If 14

# of 15 bases have 97% or better quality, then the window stops

# and the sequence before the window is cut and the bases are

# finally trimmed one by one for 97% quality.

<sequence-trim-quality-start>

title = Start quality trim

window-length = 15

window-match = 14

minimum-quality = 97

</sequence-trim-quality-start>

# Quality trimming, sequence end. We chose a 35 long window with

# rather high strictness (34 out of 35) but only 96% required,

# because end qualities tail off more gradually.

<sequence-trim-quality-end>

title = End quality trim

window-length = 35

window-match = 34

minimum-quality = 96

</sequence-trim-quality-end>

# Filter by length, minimum 200 required,

<sequence-filter>

title = Length filter

minimum-length = 200

</sequence-filter>

# Filter by overall quality, 90% of all positions must have at least

# 95% quality,

<sequence-filter-quality>

title = Quality filter

minimum-quality = 95

minimum-strict = 90

</sequence-filter-quality>

</sequence-cleaning>

# -------------------------------------------------------------------------

# SEQUENCE DEREPLICATION

# -------------------------------------------------------------------------

# This just collapses multiple identical reads into one, while keeping

# track of the read counts,

<sequence-dereplication>

title = Sequence de-replication

keep-outputs = yes

</sequence-dereplication>

# -------------------------------------------------------------------------

# CHIMERA FILTERING

# -------------------------------------------------------------------------

# The dataset is all RDP sequences (the most sequences at submission time)

# that span the *E. coli* amplicon positions 44-534, but clustered to 99%.

# The minimum score of 35 is the default, which is neither conservative or

# stringent. The method is summarized in Supplementary Materials.

<sequence-chimera-filter>

dataset-name = RDP_SSU_44-534-C99

title = Chimera filtering

word-length = 8

step-length = 4

minimum-score = 35

debug-output = yes

</sequence-chimera-filter>

# -------------------------------------------------------------------------

# SILVA PROFILE

# -------------------------------------------------------------------------

# The dataset is all Silva sequences with an assigned species name that

# span the *E. coli* 44-535 amplicon region. Word length (see Supplementary Materials

# for method summary) is 8 and every sequence oligo is used (step length

# is one). Positions with qualities below 93% were ignored. A minimum match

# of 40 oligo-percent (85-95 base-percent) was required, only the top 1%

# was requested, and non-canonical bases were skipped over. Sequences were

# matched in both directions as we did not know their orientation.

<sequence-similarities-simrank>

title = Silva similarities

input-step = sequence-chimera-filter

output-name = org_seqs_silva

dataset-name = Silva_SSU_44-534-S

match-word-length = 8

match-step-length = 1

quality-type = Sanger

minimum-base-quality = 93%

match-minimum = 40%

match-top-range = 1%

match-agct-only = yes

match-forward = yes

match-reverse = yes

# keep-outputs = no

</sequence-similarities-simrank>

# The dataset is all Silva sequences with an assigned species name that

# span the *E. coli* 44-535 amplicon region. Only similarities from sequences with

# at least 180 oligos were mapped to the Silva taxonomy. The similarities

# used from the step above must be least 40% and only the highest of these

# are used (match-use-range = 0).

<organism-taxonomy-profiler>

title = Silva taxonomy mapping

output-name = org_profile_silva

dataset-name = Silva_SSU_44-534-S

minimum-oligo-count = 180

match-minimum = 40%

match-use-range = 0%

# keep-outputs = no

</organism-taxonomy-profiler>

# Format tables, include all rows,

<organism-profile-format>

title = Silva profiles

output-name = org_profile_silva

taxonomy-minimum-score = 1

table-title-regex = (PCR)

</organism-profile-format>

# -------------------------------------------------------------------------

# RDP PROFILE

# -------------------------------------------------------------------------

# The dataset is all RDP sequences with an assigned species name that

# span the *E. coli* 44-535 amplicon region. Word length (see Supplementary Materials

# for method summary) is 8 and every sequence oligo is used (step length

# is one). Positions with qualities below 93% were ignored. A minimum match

# of 40 oligo-percent (85-95 base-percent) was required, only the top 1%

# was requested, and non-canonical bases were skipped over. Sequences were

# matched in both directions as we did not know their orientation.

<sequence-similarities-simrank>

title = RDP similarities

input-step = sequence-chimera-filter

output-name = org_seqs_rdp

dataset-name = RDP_SSU_44-534-S

match-word-length = 8

match-step-length = 1

quality-type = Sanger

minimum-base-quality = 93%

match-minimum = 40%

match-top-range = 1%

match-agct-only = yes

match-forward = yes

match-reverse = yes

# keep-outputs = no

</sequence-similarities-simrank>

# The dataset is all RDP sequences with an assigned species name that

# span the 44-535 amplicon region. Only similarities from sequences with

# at least 180 oligos were mapped to the RDP taxonomy. The similarities

# used from the step above must be least 40% and only the highest of these

# are used (match-use-range = 0).

<organism-taxonomy-profiler>

title = RDP taxonomy mapping

output-name = org_profile_rdp

dataset-name = RDP_SSU_44-534-S

minimum-oligo-count = 180

match-minimum = 40%

match-use-range = 0%

# keep-outputs = no

</organism-taxonomy-profiler>

# Format tables, include all rows,

<organism-profile-format>

title = RDP profile

output-name = org_profile_rdp

taxonomy-minimum-score = 1

table-title-regex = (PCR)

</organism-profile-format>

# -------------------------------------------------------------------------

# GREENGENES PROFILE

# -------------------------------------------------------------------------

# The dataset is all Greengenes sequences, October 2012 release. We could

# not create amplicon sub-databases, since the distributed alignments in

# this release has altered sequences. Word length (see Supplementary

# Materials for method summary) is 8 and every sequence oligo is used (step

# length is one). Positions with qualities below 93% were ignored. A minimum

# match of 40 oligo-percent (85-95 base-percent) was required, only the top

# 1% was requested, and non-canonical bases were skipped over. Sequences

# were matched in both directions as we did not know their orientation.

<sequence-similarities-simrank>

title = Greengenes similarities

input-step = sequence-chimera-filter

output-name = org_seqs_green

dataset-name = Green_SSU_all

match-word-length = 8

match-step-length = 1

quality-type = Sanger

minimum-base-quality = 93%

match-minimum = 40%

match-top-range = 1%

match-agct-only = yes

match-forward = yes

match-reverse = yes

# keep-outputs = no

</sequence-similarities-simrank>

# The dataset is all Greengenes. Only similarities from sequences with at

# least 180 oligos were mapped to the RDP taxonomy. The similarities used

# from the step above must be least 40% and only the highest of these

# are used (match-use-range = 0).

<organism-taxonomy-profiler>

title = Greengenes taxonomy mapping

output-name = org_profile_green

dataset-name = Green_SSU_all

minimum-oligo-count = 180

match-minimum = 40%

match-use-range = 0%

# keep-outputs = no

</organism-taxonomy-profiler>

<organism-profile-format>

title = Greengenes profile

output-name = org_profile_green

taxonomy-minimum-score = 1

table-title-regex = (PCR)

</organism-profile-format>

</recipe>
