# Supplementary material for: Characterising the Canine Oral Microbiome by Direct Sequencing of Reverse-Transcribed rRNA Molecules
Source: PLoS One. 2016 Jun 8;11(6):e0157046. doi: 10.1371/journal.pone.0157046 (PMC4898712; doi:10.1371/journal.pone.0157046)
Supplement: S3 Methods — (DOCX) [file pone.0157046.s003.docx]

**S3 Methods**. **RT-SSU rRNA analysis recipe**

# -*- html -*-

# The recipe below, which is input to the run_recipe command, consist of

# steps that are run in the order given. Each step has parameter keys and

# values that are passed on to the underlying method. Any routine or program

# with a command line interface can be made to appear as a recipe step.

<recipe>

title = 16S RT vs Silva, RDP and Greengenes

author = Niels Larsen, James McDonald

site = Danish Genome Institute, Bangor University

email = niels@genomics.dk

# ------------------------------------------------------------------------

# SFF FORMAT CONVERSION

# ------------------------------------------------------------------------

# Not used, but works. Can convert .sff files to fastq for example, which

# BION uses as default exchange format between steps.

# <sequence-conversion>

# title = Conversion to fastq

# </sequence-conversion>

# ------------------------------------------------------------------------

# CLEANING

# ------------------------------------------------------------------------

<sequence-cleaning>

title = Sequence cleaning

quality-type = Sanger

# Clip adapter, sequence start.

# Adapters and adapter fragments were repeated at both ends; we removed

# them by applying sequence-clip-pattern-start three times. The [1,0,0]

# means up to one mismatch, no deletions or insertions in the pattern

# sequence respectively. The 0...25 and 1...1 are "rubber bands" that

# allow any number of bases up to 25 to match and exactly one to match

# of any kind. Only the first 50 positions were matched against.

<sequence-clip-pattern-start>

title = Start adapter clip 1!

pattern-string = ^ 0...25 TCAGTGATACGTC[1,0,0] 1...1

search-distance = 50

</sequence-clip-pattern-start>

<sequence-clip-pattern-start>

title = Start adapter clip 2

pattern-string = ^ 0...25 TCAGTGATACGTC[1,0,0] 1...1

search-distance = 50

</sequence-clip-pattern-start>

<sequence-clip-pattern-start>

title = Start adapter clip 3

pattern-string = ^ 0...25 TCAGTGATACGTC[1,0,0] 1...1

search-distance = 50

</sequence-clip-pattern-start>

# Quality trimming, sequence start. A sliding window of length 15

# counts the number of bases with a quality of at least 97%. If 14

# of 15 bases have 97% or better quality, then the window stops

# and the sequence before the window is cut. The bases are finally

# trimmed one by one for 97% quality.

<sequence-trim-quality-start>

title = Start quality trim

window-length = 15

window-match = 14

minimum-quality = 97

</sequence-trim-quality-start>

# Quality trimming, sequence end. A sliding window of length 20

# counts the number of bases with a quality of at least 96%. If any

# of the 20 bases drop below 96% quality, then the window stops

# and the sequence before the window is cut. The bases are finally

# trimmed one by one for 96% quality. We do this before adapter

# clipping because the sequences were sometimes so poor the motifs

# below would not be recognized near ends.

<sequence-trim-quality-end>

title = End quality trim

window-length = 20

window-match = 20

minimum-quality = 96

</sequence-trim-quality-end>

# Clip adapter, sequence end.

# Adapters and adapter fragments were repeated at both ends; we remove

# them by applying sequence-clip-pattern-start three times. The [2,1,1]

# means up to two mismatches, and up to one deletion and insertion

# respectively, in the pattern sequence. The i...j ranges are "rubber

# bands" that allow between i and j number bases, of any kind, to

# match.

<sequence-clip-pattern-end>

title = End adapter clip 1

pattern-string = 20...20 ACACAGGGGATAGG[2,1,1] 0...20 $

search-distance = 50

</sequence-clip-pattern-end>

<sequence-clip-pattern-end>

title = End adapter clip 2

pattern-string = 8...8 TGCCAAGGCACACA[2,1,1] 0...30 $

search-distance = 50

</sequence-clip-pattern-end>

<sequence-clip-pattern-end>

title = End adapter clip 3

pattern-string = CTGAGACTGCCAAG[2,1,1] 0...50 $

search-distance = 100

</sequence-clip-pattern-end>

# Quality trimming, sequence start. A sliding window of length 15

# counts the number of bases with a quality of at least 96%. If 14

# of 15 bases have 96% or better quality, then the window stops

# and the sequence before the window is cut and the bases are

# finally trimmed one by one for 96% quality.

<sequence-trim-quality-end>

title = End quality trim

window-length = 15

window-match = 14

minimum-quality = 96

</sequence-trim-quality-end>

# Sequence trimming, sequence end. Starting at 50 positions from

# the end, the similarity between the adapter sequence and the query

# is measured. If it is 80% or better, then the window stops

# and the sequence is cut where the match starts. If not 80% or

# better, the sequence slides towards the end and past it, so there

# is less and less overlap. The query is cut at the first match of

# 80% or better. Most often this causes no bases to be cut, or just

# the last one (and the last one is dispensible, as following

# clustering steps usually will recover it).

<sequence-trim-end>

title = End sequence trim

sequence = CTGAGACTGCCAAGGCACACAGGGGATAGG

search-distance = 50

minimum-length = 1

minimum-strict = 80%

</sequence-trim-end>

# Filter by length, minimum 200 required,

<sequence-filter>

title = Length filter

minimum-length = 200

</sequence-filter>

# Filter by overall quality, 90% of all positions must have at least

# 95% quality,

<sequence-filter-quality>

title = Quality filter

minimum-quality = 95

minimum-strict = 90

</sequence-filter-quality>

# Filter away sequences with triplets that occur many times,

<sequence-filter>

title = 3-repeat filter

pattern-string-nomatch = p1=3...3 p1 p1 p1 p1 p1 p1 p1 p1 p1 p1 p1 p1 p1 p1 p1 p1

forward = yes

</sequence-filter>

# Filter away sequences with 4-mers that occur repeatedly,

<sequence-filter>

title = 4-repeat filter

pattern-string-nomatch = p1=4...4 p1 p1 p1 p1 p1 p1 p1 p1 p1 p1 p1 p1

forward = yes

</sequence-filter>

</sequence-cleaning>

# -------------------------------------------------------------------------

# SEQUENCE DEREPLICATION

# -------------------------------------------------------------------------

# This just collapses multiple identical reads into one, while keeping

# track of the read counts,

<sequence-dereplication>

title = Sequence de-replication

keep-outputs = yes

</sequence-dereplication>

# -------------------------------------------------------------------------

# CHIMERA FILTERING

# -------------------------------------------------------------------------

# The dataset is all RDP sequences (the most sequences at submission time)

# that are at least 1250 bases long. There were no amplicons in the RT dataset.

# The minimum score of 35 is the default, which is neither conservative or

# stringent. The method is summarized in Supplementary Materials.

<sequence-chimera-filter>

dataset-name = RDP_SSU_minlen_1250-S

title = Chimera filtering

word-length = 8

step-length = 4

minimum-score = 35

debug-output = yes

</sequence-chimera-filter>

# -------------------------------------------------------------------------

# SILVA PROFILE

# -------------------------------------------------------------------------

# The dataset is all Silva sequences 1250 or longer and with an assigned

# species name. Word length (see Supplementary Materials for method summary)

# is 8 and every sequence oligo is used (step length is one). Positions with

# qualities below 93% were ignored. A minimum match of 40 oligo-percent

# (85-95 base-percent) was required, only the top 1% was requested, and

# non-canonical bases were skipped over. Sequences were matched in both

# directions as with this dataset we cannot know their orientation.

<sequence-similarities-simrank>

title = Silva similarities

input-step = sequence-chimera-filter

output-name = org_seqs_silva

dataset-name = Silva_SSU_minlen_1250-S

match-word-length = 8

match-step-length = 1

quality-type = Sanger

minimum-base-quality = 93%

match-minimum = 40%

match-top-range = 1%

match-agct-only = yes

match-forward = yes

match-reverse = yes

# keep-outputs = no

</sequence-similarities-simrank>

# The dataset is all Silva sequences 1250 or longer and with an assigned

# species name. Only similarities from sequences with at least 180 oligos

# were mapped to the Silva taxonomy. The similarities used from the step

# above must be least 40% and only the highest of these are used

# (match-use-range = 0).

<organism-taxonomy-profiler>

title = Silva taxonomy mapping

output-name = org_profile_silva

dataset-name = Silva_SSU_minlen_1250-S

minimum-oligo-count = 180

match-minimum = 40%

match-use-range = 0%

# keep-outputs = no

</organism-taxonomy-profiler>

# Format tables, include all rows,

<organism-profile-format>

title = Silva profiles

output-name = org_profile

taxonomy-minimum-score = 1

table-title-regex = (RT)

</organism-profile-format>

# -------------------------------------------------------------------------

# RDP PROFILE

# -------------------------------------------------------------------------

# The dataset is all RDP sequences 1250 or longer and with an assigned

# species name. Word length (see Supplementary Materials for method summary)

# is 8 and every sequence oligo is used (step length is one). Positions with

# qualities below 93% were ignored. A minimum match of 40 oligo-percent

# (85-95 base-percent) was required, only the top 1% was requested, and

# non-canonical bases were skipped over. Sequences were matched in both

# directions as with this dataset we cannot know their orientation.

<sequence-similarities-simrank>

title = RDP similarities

input-step = sequence-chimera-filter

output-name = org_seqs_rdp

dataset-name = RDP_SSU_minlen_1250-S

match-word-length = 8

match-step-length = 1

quality-type = Sanger

minimum-base-quality = 93%

match-minimum = 40%

match-top-range = 1%

match-agct-only = yes

match-forward = yes

match-reverse = yes

# keep-outputs = no

</sequence-similarities-simrank>

# The dataset is all RDP sequences 1250 or longer and with an assigned

# species name. Only similarities from sequences with at least 180 oligos

# were mapped to the RDP taxonomy. The similarities used from the step

# above must be least 40% and only the highest of these are used

# (match-use-range = 0).

<organism-taxonomy-profiler>

title = RDP taxonomy mapping

output-name = org_profile_rdp

dataset-name = RDP_SSU_minlen_1250-S

minimum-oligo-count = 180

match-minimum = 40%

match-use-range = 0%

# keep-outputs = no

</organism-taxonomy-profiler>

# Format tables, include all rows,

<organism-profile-format>

title = RDP profiles

output-name = org_profile_rdp

taxonomy-minimum-score = 1

table-title-regex = (RT)

</organism-profile-format>

# -------------------------------------------------------------------------

# GREENGENES PROFILE

# -------------------------------------------------------------------------

# The dataset is all Greengenes sequences 1250 or longer. Word length (see

# Supplementary Materials for method summary) is 8 and every sequence oligo

# is used (step length is one). Positions with qualities below 93% were

# ignored. A minimum match of 40 oligo-percent (85-95 base-percent) was

# required, only the top 1% was requested, and non-canonical bases were

# skipped over. Sequences were matched in both directions as with this

# dataset we cannot know their orientation.

<sequence-similarities-simrank>

title = Greengenes similarities

input-step = sequence-chimera-filter

output-name = org_seqs_green

dataset-name = Green_SSU_all

match-word-length = 8

match-step-length = 1

quality-type = Sanger

minimum-base-quality = 93%

match-minimum = 40%

match-top-range = 1%

match-agct-only = yes

match-forward = yes

match-reverse = yes

# keep-outputs = no

</sequence-similarities-simrank>

# The dataset is all Greengenes sequences 1250 or longer. Only similarities

# from sequences with at least 180 oligos were mapped to the Greengenes taxonomy.

# The similarities used from the step above must be least 40% and only the

# highest of these are used (match-use-range = 0).

<organism-taxonomy-profiler>

title = Greengenes taxonomy mapping

output-name = org_profile_green

dataset-name = Green_SSU_all

minimum-oligo-count = 180

match-minimum = 40%

match-use-range = 0%

# keep-outputs = no

</organism-taxonomy-profiler>

# Format tables, include all rows,

<organism-profile-format>

title = Greengenes profile

input-step = organism-taxonomy-profiler

output-name = org_profile_green

taxonomy-minimum-score = 1

table-title-regex = (RT)

</organism-profile-format>

</recipe>
