# Supplementary material for: Characterising the Canine Oral Microbiome by Direct Sequencing of Reverse-Transcribed rRNA Molecules
Source: PLoS One. 2016 Jun 8;11(6):e0157046. doi: 10.1371/journal.pone.0157046 (PMC4898712; doi:10.1371/journal.pone.0157046)
Supplement: S1 Table — (DOCX) [file pone.0157046.s004.docx]

**S1 Table. Summary table of statistics for the processing and classification of the sequence data presented in Fig. 2.**

|  | 16S rRNA gene PCR amplicons | | RT-SSU rRNA sequences | |
| --- | --- | --- | --- | --- |
|  | **Qiime** | **BION-meta** | **RDP classifier** | **BION-meta** |
| **Sequences in raw dataset** | 248,760 | 248,760 | 257,043 | 257,043 |
| **Sequences remaining following QC** | 149,185 | 172,188^a^ | 211,559 | 225,023^a^ |
| **Sequences remaining following chimera removal** | 153,866^b^ | 167,823 | N/A | 221,172 |
| **Classified sequences** | 67,682 | 166,191 | 115,699, | 111,010 |
| **% sequences classified** | 27% | 67% | 45% | 43% |

**^a^** Most sequences are removed because they are short (<200 bp) or, some because they are low quality (90% of all positions must have at least 95% quality values) (Supplementary Methods 2). Please refer to ‘Supplementary Methods 1’ for description of BION-meta. ^b^ The chimera removal step was completed prior to sequence QC, hence the higher number of sequences shown at this step.
