# Supplementary material for: Characterising the Canine Oral Microbiome by Direct Sequencing of Reverse-Transcribed rRNA Molecules
Source: PLoS One. 2016 Jun 8;11(6):e0157046. doi: 10.1371/journal.pone.0157046 (PMC4898712; doi:10.1371/journal.pone.0157046)
Supplement: S2 Table — (DOCX) [file pone.0157046.s005.docx]

**S2 Table. Classification of 16S rRNA gene PCR amplicons (Accession: SRR830918) and RT-SSU rRNA sequence reads (Accession: SRR830919) using the RDP classifier and library comparison tool.**

|  |  |  |  |  |  |  |  |
| --- | --- | --- | --- | --- | --- | --- | --- |
|  | **Taxon** | **Rank** | **RT-SSU rRNA sequencing** | | **16S rRNA gene PCR amplicons** | |  |
|  |  |  | **Read number** | **% of total** | **Read number** | **% of total** |  |
|  |  |  |  |  |  |  |  |
|  | **Bacteria** | **domain** | **211559** | **100.0000** | **231126** | **100.0000** |  |
|  |  |  |  |  |  |  |  |
|  | **Proteobacteria** | **phylum** | **62080** | **29.3441** | **40479** | **17.5138** |  |
|  | Gammaproteobacteria | class | 33095 | 15.6434 | 22301 | 9.6488 |  |
|  | Pseudomonadales | order | 24868 | 11.7546 | 7859 | 3.4003 |  |
|  | Moraxellaceae | family | 24780 | 11.7130 | 7691 | 3.3276 |  |
|  | Enhydrobacter | genus | 9343 | 4.4163 | 3441 | 1.4888 |  |
|  | Moraxella | genus | 6901 | 3.2620 | 2025 | 0.8761 |  |
|  | Acinetobacter | genus | 141 | 0.0666 | 204 | 0.0883 |  |
|  | Psychrobacter | genus | 13 | 0.0061 | 2 | 0.0009 |  |
|  | unclassified_Moraxellaceae |  | 8382 | 3.9620 | 2019 | 0.8735 |  |
|  | Xanthomonadales | order | 2799 | 1.3230 | 4816 | 2.0837 |  |
|  | Xanthomonadaceae | family | 2796 | 1.3216 | 4813 | 2.0824 |  |
|  | Thermomonas | genus | 160 | 0.0756 | 0 | 0.0000 |  |
|  | Lysobacter | genus | 109 | 0.0515 | 840 | 0.3634 |  |
|  | Xanthomonas | genus | 14 | 0.0066 | 19 | 0.0082 |  |
|  | Dyella | genus | 6 | 0.0028 | 0 | 0.0000 |  |
|  | Luteimonas | genus | 21 | 0.0099 | 1 | 0.0004 |  |
|  | Stenotrophomonas | genus | 3 | 0.0014 | 13 | 0.0056 |  |
|  | Dokdonella | genus | 4 | 0.0019 | 0 | 0.0000 |  |
|  | unclassified_Xanthomonadaceae |  | 2479 | 1.1718 | 3940 | 1.7047 |  |
|  | Pasteurellales | order | 1474 | 0.6967 | 5097 | 2.2053 |  |
|  | Pasteurellaceae | family | 1474 | 0.6967 | 5097 | 2.2053 |  |
|  | Pasteurella | genus | 330 | 0.1560 | 2223 | 0.9618 |  |
|  | Haemophilus | genus | 55 | 0.0260 | 139 | 0.0601 |  |
|  | Actinobacillus | genus | 119 | 0.0562 | 142 | 0.0614 |  |
|  | Lonepinella | genus | 1 | 0.0005 | 0 | 0.0000 |  |
|  | Bibersteinia | genus | 2 | 0.0009 | 35 | 0.0151 |  |
|  | Aggregatibacter | genus | 0 | 0.0000 | 10 | 0.0043 |  |
|  | Mannheimia | genus | 1 | 0.0005 | 1 | 0.0004 |  |
|  | Avibacterium | genus | 1 | 0.0005 | 0 | 0.0000 |  |
|  | unclassified_Pasteurellaceae |  | 965 | 0.4561 | 2547 | 1.1020 |  |
|  | Cardiobacteriales | order | 737 | 0.3484 | 304 | 0.1315 |  |
|  | Cardiobacteriaceae | family | 737 | 0.3484 | 304 | 0.1315 |  |
|  | Cardiobacterium | genus | 141 | 0.0666 | 4 | 0.0017 |  |
|  | Suttonella | genus | 284 | 0.1342 | 11 | 0.0048 |  |
|  | unclassified_Cardiobacteriaceae |  | 312 | 0.1475 | 289 | 0.1250 |  |
|  | Alteromonadales | order | 10 | 0.0047 | 0 | 0.0000 |  |
|  | Shewanellaceae | family | 9 | 0.0043 | 0 | 0.0000 |  |
|  | Shewanella | genus | 9 | 0.0043 | 0 | 0.0000 |  |
|  | unclassified_Pseudomonadales |  | 88 | 0.0416 | 168 | 0.0727 |  |
|  | Enterobacteriales | order | 0 | 0.0000 | 3 | 0.0013 |  |
|  | Enterobacteriaceae | family | 0 | 0.0000 | 3 | 0.0013 |  |
|  | Escherichia/Shigella | genus | 0 | 0.0000 | 1 | 0.0004 |  |
|  | unclassified_Enterobacteriaceae |  | 0 | 0.0000 | 2 | 0.0009 |  |
|  | unclassified_Xanthomonadales |  | 3 | 0.0014 | 3 | 0.0013 |  |
|  | unclassified_Alteromonadales |  | 1 | 0.0005 | 0 | 0.0000 |  |
|  | Aeromonadales | order | 1 | 0.0005 | 0 | 0.0000 |  |
|  | Succinivibrionaceae | family | 1 | 0.0005 | 0 | 0.0000 |  |
|  | Anaerobiospirillum | genus | 1 | 0.0005 | 0 | 0.0000 |  |
|  | Epsilonproteobacteria | class | 4207 | 1.9886 | 801 | 0.3466 |  |
|  | Campylobacterales | order | 4184 | 1.9777 | 792 | 0.3427 |  |
|  | Campylobacteraceae | family | 3782 | 1.7877 | 270 | 0.1168 |  |
|  | Campylobacter | genus | 2055 | 0.9714 | 171 | 0.0740 |  |
|  | Arcobacter | genus | 1718 | 0.8121 | 92 | 0.0398 |  |
|  | Helicobacteraceae | family | 365 | 0.1725 | 514 | 0.2224 |  |
|  | Wolinella | genus | 315 | 0.1489 | 433 | 0.1873 |  |
|  | Helicobacter | genus | 6 | 0.0028 | 1 | 0.0004 |  |
|  | unclassified_Campylobacteraceae |  | 9 | 0.0043 | 7 | 0.0030 |  |
|  | unclassified_Helicobacteraceae |  | 44 | 0.0208 | 8 | 0.0035 |  |
|  | unclassified_Campylobacterales |  | 37 | 0.0175 | 8 | 0.0035 |  |
|  | Nautiliales | order | 3 | 0.0014 | 0 | 0.0000 |  |
|  | Nautiliaceae | family | 3 | 0.0014 | 0 | 0.0000 |  |
|  | Thioreductor | genus | 3 | 0.0014 | 0 | 0.0000 |  |
|  | unclassified_Gammaproteobacteria |  | 3206 | 1.5154 | 4222 | 1.8267 |  |
|  | Betaproteobacteria | class | 22030 | 10.4132 | 10939 | 4.7329 |  |
|  | Burkholderiales | order | 14938 | 7.0609 | 7631 | 3.3017 |  |
|  | Comamonadaceae | family | 12549 | 5.9317 | 5097 | 2.2053 |  |
|  | Hydrogenophaga | genus | 15 | 0.0071 | 0 | 0.0000 |  |
|  | Ottowia | genus | 27 | 0.0128 | 181 | 0.0783 |  |
|  | Variovorax | genus | 389 | 0.1839 | 0 | 0.0000 |  |
|  | Comamonas | genus | 647 | 0.3058 | 36 | 0.0156 |  |
|  | Brachymonas | genus | 210 | 0.0993 | 1003 | 0.4340 |  |
|  | Lampropedia | genus | 475 | 0.2245 | 19 | 0.0082 |  |
|  | Tepidicella | genus | 3 | 0.0014 | 0 | 0.0000 |  |
|  | Xenophilus | genus | 14 | 0.0066 | 0 | 0.0000 |  |
|  | Schlegelella | genus | 1 | 0.0005 | 3 | 0.0013 |  |
|  | Acidovorax | genus | 4 | 0.0019 | 1 | 0.0004 |  |
|  | Delftia | genus | 2 | 0.0009 | 3 | 0.0013 |  |
|  | unclassified_Comamonadaceae |  | 10762 | 5.0870 | 3851 | 1.6662 |  |
|  | Alcaligenaceae | family | 374 | 0.1768 | 774 | 0.3349 |  |
|  | Castellaniella | genus | 0 | 0.0000 | 2 | 0.0009 |  |
|  | Advenella | genus | 48 | 0.0227 | 33 | 0.0143 |  |
|  | Parapusillimonas | genus | 0 | 0.0000 | 1 | 0.0004 |  |
|  | Pigmentiphaga | genus | 0 | 0.0000 | 2 | 0.0009 |  |
|  | Alcaligenes | genus | 0 | 0.0000 | 1 | 0.0004 |  |
|  | Achromobacter | genus | 2 | 0.0009 | 12 | 0.0052 |  |
|  | Kerstersia | genus | 0 | 0.0000 | 1 | 0.0004 |  |
|  | unclassified_Alcaligenaceae |  | 324 | 0.1531 | 722 | 0.3124 |  |
|  | Oxalobacteraceae | family | 21 | 0.0099 | 1 | 0.0004 |  |
|  | Oxalobacter | genus | 0 | 0.0000 | 1 | 0.0004 |  |
|  | unclassified_Oxalobacteraceae |  | 21 | 0.0099 | 0 | 0.0000 |  |
|  | Burkholderiaceae | family | 13 | 0.0061 | 42 | 0.0182 |  |
|  | Burkholderia | genus | 2 | 0.0009 | 1 | 0.0004 |  |
|  | Pandoraea | genus | 7 | 0.0033 | 14 | 0.0061 |  |
|  | unclassified_Burkholderiaceae |  | 4 | 0.0019 | 27 | 0.0117 |  |
|  | Sutterellaceae | family | 1 | 0.0005 | 2 | 0.0009 |  |
|  | Sutterella | genus | 1 | 0.0005 | 1 | 0.0004 |  |
|  | Parasutterella | genus | 0 | 0.0000 | 1 | 0.0004 |  |
|  | Burkholderiales_incertae_sedis | family | 1 | 0.0005 | 0 | 0.0000 |  |
|  | unclassified_Burkholderiales_incertae_sedis | | 1 | 0.0005 | 0 | 0.0000 |  |
|  | unclassified_Burkholderiales |  | 1979 | 0.9354 | 1715 | 0.7420 |  |
|  | Neisseriales | order | 5905 | 2.7912 | 2359 | 1.0207 |  |
|  | Neisseriaceae | family | 5905 | 2.7912 | 2359 | 1.0207 |  |
|  | Neisseria | genus | 1436 | 0.6788 | 1520 | 0.6576 |  |
|  | Aquaspirillum | genus | 1054 | 0.4982 | 0 | 0.0000 |  |
|  | Vogesella | genus | 4 | 0.0019 | 0 | 0.0000 |  |
|  | Conchiformibius | genus | 33 | 0.0156 | 19 | 0.0082 |  |
|  | Kingella | genus | 2 | 0.0009 | 10 | 0.0043 |  |
|  | Alysiella | genus | 6 | 0.0028 | 1 | 0.0004 |  |
|  | Stenoxybacter | genus | 1 | 0.0005 | 0 | 0.0000 |  |
|  | unclassified_Neisseriaceae |  | 3369 | 1.5925 | 809 | 0.3500 |  |
|  | Rhodocyclales | order | 99 | 0.0468 | 277 | 0.1198 |  |
|  | Rhodocyclaceae | family | 99 | 0.0468 | 277 | 0.1198 |  |
|  | Propionivibrio | genus | 43 | 0.0203 | 28 | 0.0121 |  |
|  | Azospira | genus | 0 | 0.0000 | 4 | 0.0017 |  |
|  | unclassified_Rhodocyclaceae |  | 56 | 0.0265 | 245 | 0.1060 |  |
|  | unclassified_Epsilonproteobacteria |  | 20 | 0.0095 | 9 | 0.0039 |  |
|  | Deltaproteobacteria | class | 1365 | 0.6452 | 3608 | 1.5611 |  |
|  | Myxococcales | order | 110 | 0.0520 | 26 | 0.0112 |  |
|  | Sorangiineae | suborder | 99 | 0.0468 | 25 | 0.0108 |  |
|  | Polyangiaceae | family | 85 | 0.0402 | 24 | 0.0104 |  |
|  | Sorangium | genus | 26 | 0.0123 | 8 | 0.0035 |  |
|  | Byssovorax | genus | 1 | 0.0005 | 0 | 0.0000 |  |
|  | unclassified_Polyangiaceae |  | 58 | 0.0274 | 16 | 0.0069 |  |
|  | unclassified_Sorangiineae |  | 14 | 0.0066 | 1 | 0.0004 |  |
|  | Desulfovibrionales | order | 1048 | 0.4954 | 2857 | 1.2361 |  |
|  | Desulfomicrobiaceae | family | 892 | 0.4216 | 2382 | 1.0306 |  |
|  | Desulfomicrobium | genus | 892 | 0.4216 | 2382 | 1.0306 |  |
|  | Desulfovibrionaceae | family | 113 | 0.0534 | 354 | 0.1532 |  |
|  | Desulfovibrio | genus | 32 | 0.0151 | 3 | 0.0013 |  |
|  | Bilophila | genus | 0 | 0.0000 | 1 | 0.0004 |  |
|  | unclassified_Desulfovibrionaceae |  | 81 | 0.0383 | 350 | 0.1514 |  |
|  | Bdellovibrionales | order | 134 | 0.0633 | 329 | 0.1423 |  |
|  | Bdellovibrionaceae | family | 131 | 0.0619 | 329 | 0.1423 |  |
|  | Bdellovibrio | genus | 131 | 0.0619 | 329 | 0.1423 |  |
|  | Bacteriovoracaceae | family | 3 | 0.0014 | 0 | 0.0000 |  |
|  | Bacteriovorax | genus | 1 | 0.0005 | 0 | 0.0000 |  |
|  | unclassified_Bacteriovoracaceae |  | 2 | 0.0009 | 0 | 0.0000 |  |
|  | unclassified_Desulfovibrionales |  | 43 | 0.0203 | 121 | 0.0524 |  |
|  | Desulfobacterales | order | 12 | 0.0057 | 56 | 0.0242 |  |
|  | Desulfobulbaceae | family | 12 | 0.0057 | 56 | 0.0242 |  |
|  | Desulfobulbus | genus | 11 | 0.0052 | 56 | 0.0242 |  |
|  | unclassified_Desulfobulbaceae |  | 1 | 0.0005 | 0 | 0.0000 |  |
|  | unclassified_Myxococcales |  | 11 | 0.0052 | 1 | 0.0004 |  |
|  | unclassified_Betaproteobacteria |  | 1088 | 0.5143 | 672 | 0.2908 |  |
|  | unclassified_Deltaproteobacteria |  | 61 | 0.0288 | 340 | 0.1471 |  |
|  | Alphaproteobacteria | class | 12 | 0.0057 | 68 | 0.0294 |  |
|  | Caulobacterales | order | 0 | 0.0000 | 34 | 0.0147 |  |
|  | Caulobacteraceae | family | 0 | 0.0000 | 31 | 0.0134 |  |
|  | Brevundimonas | genus | 0 | 0.0000 | 26 | 0.0112 |  |
|  | Caulobacter | genus | 0 | 0.0000 | 1 | 0.0004 |  |
|  | Phenylobacterium | genus | 0 | 0.0000 | 1 | 0.0004 |  |
|  | Sphingomonadales | order | 1 | 0.0005 | 0 | 0.0000 |  |
|  | Sphingomonadaceae | family | 1 | 0.0005 | 0 | 0.0000 |  |
|  | Sphingomonas | genus | 1 | 0.0005 | 0 | 0.0000 |  |
|  | Rhizobiales | order | 1 | 0.0005 | 29 | 0.0125 |  |
|  | Phyllobacteriaceae | family | 0 | 0.0000 | 5 | 0.0022 |  |
|  | Aquamicrobium | genus | 0 | 0.0000 | 5 | 0.0022 |  |
|  | Brucellaceae | family | 0 | 0.0000 | 15 | 0.0065 |  |
|  | Ochrobactrum | genus | 0 | 0.0000 | 15 | 0.0065 |  |
|  | unclassified_Caulobacterales |  | 0 | 0.0000 | 3 | 0.0013 |  |
|  | unclassified_Rhizobiales |  | 0 | 0.0000 | 9 | 0.0039 |  |
|  | Hyphomicrobiaceae | family | 1 | 0.0005 | 0 | 0.0000 |  |
|  | Maritalea | genus | 1 | 0.0005 | 0 | 0.0000 |  |
|  | unclassified_Alphaproteobacteria |  | 10 | 0.0047 | 5 | 0.0022 |  |
|  |  |  |  |  |  |  |  |
|  | **Spirochaetes** | **phylum** | **8920** | **4.2163** | **640** | **0.2769** |  |
|  | Spirochaetes | class | 8920 | 4.2163 | 640 | 0.2769 |  |
|  | Spirochaetales | order | 8920 | 4.2163 | 640 | 0.2769 |  |
|  | Spirochaetaceae | family | 8870 | 4.1927 | 639 | 0.2765 |  |
|  | Treponema | genus | 8796 | 4.1577 | 636 | 0.2752 |  |
|  | Spirochaeta | genus | 14 | 0.0066 | 0 | 0.0000 |  |
|  | unclassified_Spirochaetaceae |  | 60 | 0.0284 | 3 | 0.0013 |  |
|  | unclassified_Spirochaetales |  | 50 | 0.0236 | 1 | 0.0004 |  |
|  |  |  |  |  |  |  |  |
|  | **Bacteroidetes** | **phylum** | **28205** | **13.3320** | **122122** | **52.8378** |  |
|  | Bacteroidia | class | 16705 | 7.8961 | 109217 | 47.2543 |  |
|  | Bacteroidales | order | 16705 | 7.8961 | 109217 | 47.2543 |  |
|  | Porphyromonadaceae | family | 14471 | 6.8402 | 102376 | 44.2945 |  |
|  | Porphyromonas | genus | 12422 | 5.8716 | 95106 | 41.1490 |  |
|  | Tannerella | genus | 549 | 0.2595 | 907 | 0.3924 |  |
|  | Paludibacter | genus | 29 | 0.0137 | 27 | 0.0117 |  |
|  | Parabacteroides | genus | 55 | 0.0260 | 302 | 0.1307 |  |
|  | Proteiniphilum | genus | 11 | 0.0052 | 109 | 0.0472 |  |
|  | Odoribacter | genus | 13 | 0.0061 | 28 | 0.0121 |  |
|  | Petrimonas | genus | 3 | 0.0014 | 12 | 0.0052 |  |
|  | Dysgonomonas | genus | 0 | 0.0000 | 4 | 0.0017 |  |
|  | Prevotellaceae | family | 591 | 0.2794 | 1559 | 0.6745 |  |
|  | Paraprevotella | genus | 64 | 0.0303 | 707 | 0.3059 |  |
|  | Prevotella | genus | 34 | 0.0161 | 65 | 0.0281 |  |
|  | Hallella | genus | 5 | 0.0024 | 6 | 0.0026 |  |
|  | unclassified_"Porphyromonadaceae" |  | 1389 | 0.6566 | 5881 | 2.5445 |  |
|  | Bacteroidaceae | family | 458 | 0.2165 | 410 | 0.1774 |  |
|  | Bacteroides | genus | 450 | 0.2127 | 409 | 0.1770 |  |
|  | Anaerorhabdus | genus | 8 | 0.0038 | 1 | 0.0004 |  |
|  | unclassified_"Prevotellaceae" |  | 488 | 0.2307 | 781 | 0.3379 |  |
|  | Rikenellaceae | family | 16 | 0.0076 | 0 | 0.0000 |  |
|  | Rikenella | genus | 16 | 0.0076 | 0 | 0.0000 |  |
|  | unclassified_"Bacteroidales" |  | 1169 | 0.5526 | 4872 | 2.1079 |  |
|  | Flavobacteria | class | 7697 | 3.6382 | 3175 | 1.3737 |  |
|  | Flavobacteriales | order | 7697 | 3.6382 | 3175 | 1.3737 |  |
|  | Flavobacteriaceae | family | 7689 | 3.6344 | 3145 | 1.3607 |  |
|  | Capnocytophaga | genus | 5466 | 2.5837 | 959 | 0.4149 |  |
|  | Bergeyella | genus | 1028 | 0.4859 | 767 | 0.3319 |  |
|  | Flavobacterium | genus | 154 | 0.0728 | 661 | 0.2860 |  |
|  | Myroides | genus | 0 | 0.0000 | 1 | 0.0004 |  |
|  | Chryseobacterium | genus | 22 | 0.0104 | 0 | 0.0000 |  |
|  | Maribacter | genus | 1 | 0.0005 | 0 | 0.0000 |  |
|  | Tenacibaculum | genus | 3 | 0.0014 | 0 | 0.0000 |  |
|  | unclassified_Flavobacteriaceae |  | 1015 | 0.4798 | 757 | 0.3275 |  |
|  | unclassified_"Flavobacteriales" |  | 8 | 0.0038 | 30 | 0.0130 |  |
|  | Sphingobacteria | class | 7 | 0.0033 | 20 | 0.0087 |  |
|  | Sphingobacteriales | order | 7 | 0.0033 | 20 | 0.0087 |  |
|  | Chitinophagaceae | family | 1 | 0.0005 | 0 | 0.0000 |  |
|  | Terrimonas | genus | 1 | 0.0005 | 0 | 0.0000 |  |
|  | Sphingobacteriaceae | family | 1 | 0.0005 | 1 | 0.0004 |  |
|  | Pedobacter | genus | 1 | 0.0005 | 0 | 0.0000 |  |
|  | unclassified_Sphingobacteriaceae |  | 0 | 0.0000 | 1 | 0.0004 |  |
|  | unclassified_"Sphingobacteriales" |  | 5 | 0.0024 | 19 | 0.0082 |  |
|  | unclassified_"Bacteroidetes" |  | 0 | 0.0000 | 9710 | 4.2012 |  |
|  |  |  |  |  |  |  |  |
|  | **TM7** | **phylum** | **420** | **0.1985** | **3423** | **1.4810** |  |
|  | TM7_genera_incertae_sedis | genus | 420 | 0.1985 | 3423 | 1.4810 |  |
|  |  |  |  |  |  |  |  |
|  | **SR1** | **phylum** | **1406** | **0.6646** | **8717** | **3.7715** |  |
|  | SR1_genera_incertae_sedis | genus | 1406 | 0.6646 | 8717 | 3.7715 |  |
|  |  |  |  |  |  |  |  |
|  | **Firmicutes** | **phylum** | **9697** | **4.5836** | **13610** | **5.8886** |  |
|  | Clostridia | class | 7987 | 3.7753 | 10302 | 4.4573 |  |
|  | Clostridiales | order | 7887 | 3.7280 | 10012 | 4.3318 |  |
|  | Clostridiales_Incertae Sedis XI | family | 403 | 0.1905 | 94 | 0.0407 |  |
|  | Tissierella | genus | 14 | 0.0066 | 0 | 0.0000 |  |
|  | Helcococcus | genus | 206 | 0.0974 | 0 | 0.0000 |  |
|  | Sporanaerobacter | genus | 18 | 0.0085 | 11 | 0.0048 |  |
|  | Parvimonas | genus | 51 | 0.0241 | 21 | 0.0091 |  |
|  | Peptoniphilus | genus | 1 | 0.0005 | 0 | 0.0000 |  |
|  | Clostridiales_Incertae Sedis XII | family | 150 | 0.0709 | 194 | 0.0839 |  |
|  | Fusibacter | genus | 78 | 0.0369 | 0 | 0.0000 |  |
|  | Lachnospiraceae | family | 1656 | 0.7828 | 1023 | 0.4426 |  |
|  | Catonella | genus | 555 | 0.2623 | 120 | 0.0519 |  |
|  | Dorea | genus | 0 | 0.0000 | 1 | 0.0004 |  |
|  | Blautia | genus | 1 | 0.0005 | 0 | 0.0000 |  |
|  | Clostridium XlVb | genus | 1 | 0.0005 | 0 | 0.0000 |  |
|  | Butyrivibrio | genus | 2 | 0.0009 | 0 | 0.0000 |  |
|  | Clostridium XlVa | genus | 3 | 0.0014 | 6 | 0.0026 |  |
|  | unclassified_Lachnospiraceae |  | 1094 | 0.5171 | 896 | 0.3877 |  |
|  | Peptostreptococcaceae | family | 1979 | 0.9354 | 1327 | 0.5741 |  |
|  | Filifactor | genus | 821 | 0.3881 | 1164 | 0.5036 |  |
|  | Peptostreptococcaceae_incertae_sedis | genus | 366 | 0.1730 | 22 | 0.0095 |  |
|  | Peptostreptococcus | genus | 20 | 0.0095 | 10 | 0.0043 |  |
|  | Acetoanaerobium | genus | 209 | 0.0988 | 17 | 0.0074 |  |
|  | Proteocatella | genus | 45 | 0.0213 | 28 | 0.0121 |  |
|  | Clostridium XI | genus | 4 | 0.0019 | 0 | 0.0000 |  |
|  | unclassified_Peptostreptococcaceae |  | 514 | 0.2430 | 86 | 0.0372 |  |
|  | Peptococcaceae 1 | family | 322 | 0.1522 | 366 | 0.1584 |  |
|  | Peptococcus | genus | 264 | 0.1248 | 344 | 0.1488 |  |
|  | unclassified_Clostridiales_Incertae Sedis XI |  | 113 | 0.0534 | 62 | 0.0268 |  |
|  | Ruminococcaceae | family | 59 | 0.0279 | 342 | 0.1480 |  |
|  | Flavonifractor | genus | 10 | 0.0047 | 98 | 0.0424 |  |
|  | Faecalibacterium | genus | 0 | 0.0000 | 1 | 0.0004 |  |
|  | Clostridium IV | genus | 3 | 0.0014 | 0 | 0.0000 |  |
|  | Anaerotruncus | genus | 1 | 0.0005 | 22 | 0.0095 |  |
|  | Saccharofermentans | genus | 1 | 0.0005 | 9 | 0.0039 |  |
|  | unclassified_Ruminococcaceae |  | 44 | 0.0208 | 212 | 0.0917 |  |
|  | Clostridiales_Incertae Sedis XIII | family | 86 | 0.0407 | 242 | 0.1047 |  |
|  | Anaerovorax | genus | 68 | 0.0321 | 220 | 0.0952 |  |
|  | unclassified_Clostridiales_Incertae Sedis XII | | 72 | 0.0340 | 194 | 0.0839 |  |
|  | unclassified_Peptococcaceae 1 |  | 58 | 0.0274 | 22 | 0.0095 |  |
|  | unclassified_Clostridiales_Incertae Sedis XIII | | 18 | 0.0085 | 22 | 0.0095 |  |
|  | Anaerosphaera | genus | 0 | 0.0000 | 1 | 0.0004 |  |
|  | Eubacteriaceae | family | 0 | 0.0000 | 1 | 0.0004 |  |
|  | Eubacterium | genus | 0 | 0.0000 | 1 | 0.0004 |  |
|  | Clostridiaceae 1 | family | 1 | 0.0005 | 0 | 0.0000 |  |
|  | Clostridium sensu stricto | genus | 1 | 0.0005 | 0 | 0.0000 |  |
|  | Clostridiaceae 2 | family | 2 | 0.0009 | 0 | 0.0000 |  |
|  | Alkaliphilus | genus | 2 | 0.0009 | 0 | 0.0000 |  |
|  | unclassified_Clostridiales |  | 3229 | 1.5263 | 6422 | 2.7786 |  |
|  | Bacilli | class | 606 | 0.2864 | 506 | 0.2189 |  |
|  | Lactobacillales | order | 587 | 0.2775 | 495 | 0.2142 |  |
|  | Carnobacteriaceae | family | 498 | 0.2354 | 356 | 0.1540 |  |
|  | Granulicatella | genus | 472 | 0.2231 | 340 | 0.1471 |  |
|  | Aerococcaceae | family | 17 | 0.0080 | 92 | 0.0398 |  |
|  | Abiotrophia | genus | 2 | 0.0009 | 2 | 0.0009 |  |
|  | unclassified_Aerococcaceae |  | 15 | 0.0071 | 90 | 0.0389 |  |
|  | unclassified_Carnobacteriaceae |  | 26 | 0.0123 | 16 | 0.0069 |  |
|  | Streptococcaceae | family | 24 | 0.0113 | 1 | 0.0004 |  |
|  | Streptococcus | genus | 24 | 0.0113 | 1 | 0.0004 |  |
|  | Enterococcaceae | family | 2 | 0.0009 | 0 | 0.0000 |  |
|  | Enterococcus | genus | 1 | 0.0005 | 0 | 0.0000 |  |
|  | unclassified_Enterococcaceae |  | 1 | 0.0005 | 0 | 0.0000 |  |
|  | Bacillales | order | 13 | 0.0061 | 0 | 0.0000 |  |
|  | Bacillales_Incertae Sedis XI | family | 12 | 0.0057 | 0 | 0.0000 |  |
|  | Gemella | genus | 12 | 0.0057 | 0 | 0.0000 |  |
|  | Bacillales_Incertae Sedis XII | family | 1 | 0.0005 | 0 | 0.0000 |  |
|  | Exiguobacterium | genus | 1 | 0.0005 | 0 | 0.0000 |  |
|  | unclassified_Lactobacillales |  | 46 | 0.0217 | 46 | 0.0199 |  |
|  | unclassified_Clostridia |  | 100 | 0.0473 | 290 | 0.1255 |  |
|  | Negativicutes | class | 112 | 0.0529 | 687 | 0.2972 |  |
|  | Selenomonadales | order | 112 | 0.0529 | 687 | 0.2972 |  |
|  | Veillonellaceae | family | 106 | 0.0501 | 547 | 0.2367 |  |
|  | Schwartzia | genus | 28 | 0.0132 | 299 | 0.1294 |  |
|  | Selenomonas | genus | 8 | 0.0038 | 5 | 0.0022 |  |
|  | Megamonas | genus | 1 | 0.0005 | 2 | 0.0009 |  |
|  | unclassified_Veillonellaceae |  | 69 | 0.0326 | 241 | 0.1043 |  |
|  | Acidaminococcaceae | family | 0 | 0.0000 | 37 | 0.0160 |  |
|  | Succiniclasticum | genus | 0 | 0.0000 | 5 | 0.0022 |  |
|  | unclassified_Acidaminococcaceae |  | 0 | 0.0000 | 32 | 0.0138 |  |
|  | unclassified_Firmicutes |  | 940 | 0.4443 | 2090 | 0.9043 |  |
|  | unclassified_Selenomonadales |  | 6 | 0.0028 | 103 | 0.0446 |  |
|  | Erysipelotrichia | class | 52 | 0.0246 | 25 | 0.0108 |  |
|  | Erysipelotrichales | order | 52 | 0.0246 | 25 | 0.0108 |  |
|  | Erysipelotrichaceae | family | 52 | 0.0246 | 25 | 0.0108 |  |
|  | Holdemania | genus | 18 | 0.0085 | 0 | 0.0000 |  |
|  | Allobaculum | genus | 3 | 0.0014 | 6 | 0.0026 |  |
|  | Turicibacter | genus | 2 | 0.0009 | 0 | 0.0000 |  |
|  | Solobacterium | genus | 1 | 0.0005 | 0 | 0.0000 |  |
|  | unclassified_Erysipelotrichaceae |  | 28 | 0.0132 | 19 | 0.0082 |  |
|  | unclassified_Bacilli |  | 6 | 0.0028 | 11 | 0.0048 |  |
|  | unclassified_"Bacteroidetes" |  | 3796 | 1.7943 | 0 | 0.0000 |  |
|  |  |  |  |  |  |  |  |
|  | **Actinobacteria** | **phylum** | **3884** | **1.8359** | **11990** | **5.1876** |  |
|  | Actinobacteria | class | 3884 | 1.8359 | 11990 | 5.1876 |  |
|  | Actinobacteridae | subclass | 3651 | 1.7258 | 11536 | 4.9912 |  |
|  | Actinomycetales | order | 3635 | 1.7182 | 11388 | 4.9272 |  |
|  | Corynebacterineae | suborder | 1252 | 0.5918 | 2739 | 1.1851 |  |
|  | Corynebacteriaceae | family | 1212 | 0.5729 | 2659 | 1.1505 |  |
|  | Corynebacterium | genus | 1188 | 0.5615 | 2600 | 1.1249 |  |
|  | Turicella | genus | 0 | 0.0000 | 1 | 0.0004 |  |
|  | unclassified_Corynebacteriaceae |  | 24 | 0.0113 | 58 | 0.0251 |  |
|  | Mycobacteriaceae | family | 1 | 0.0005 | 0 | 0.0000 |  |
|  | Mycobacterium | genus | 1 | 0.0005 | 0 | 0.0000 |  |
|  | unclassified_Corynebacterineae |  | 39 | 0.0184 | 80 | 0.0346 |  |
|  | Actinomycineae | suborder | 1415 | 0.6688 | 3972 | 1.7185 |  |
|  | Actinomycetaceae | family | 1415 | 0.6688 | 3972 | 1.7185 |  |
|  | Actinomyces | genus | 1352 | 0.6391 | 3825 | 1.6549 |  |
|  | unclassified_Actinomycetaceae |  | 63 | 0.0298 | 147 | 0.0636 |  |
|  | Micrococcineae | suborder | 414 | 0.1957 | 2062 | 0.8922 |  |
|  | Microbacteriaceae | family | 313 | 0.1479 | 709 | 0.3068 |  |
|  | Leucobacter | genus | 26 | 0.0123 | 67 | 0.0290 |  |
|  | Rathayibacter | genus | 0 | 0.0000 | 6 | 0.0026 |  |
|  | Pseudoclavibacter | genus | 6 | 0.0028 | 0 | 0.0000 |  |
|  | Frigoribacterium | genus | 1 | 0.0005 | 0 | 0.0000 |  |
|  | Zimmermannella | genus | 1 | 0.0005 | 0 | 0.0000 |  |
|  | unclassified_Microbacteriaceae |  | 279 | 0.1319 | 636 | 0.2752 |  |
|  | Micrococcaceae | family | 1 | 0.0005 | 3 | 0.0013 |  |
|  | Rothia | genus | 1 | 0.0005 | 3 | 0.0013 |  |
|  | Propionibacterineae | suborder | 139 | 0.0657 | 271 | 0.1173 |  |
|  | Propionibacteriaceae | family | 138 | 0.0652 | 271 | 0.1173 |  |
|  | Luteococcus | genus | 0 | 0.0000 | 1 | 0.0004 |  |
|  | Tessaracoccus | genus | 19 | 0.0090 | 2 | 0.0009 |  |
|  | Brooklawnia | genus | 2 | 0.0009 | 3 | 0.0013 |  |
|  | unclassified_Propionibacteriaceae |  | 117 | 0.0553 | 0 | 0.0000 |  |
|  | unclassified_Micrococcineae |  | 100 | 0.0473 | 1350 | 0.5841 |  |
|  | Kineosporiineae | suborder | 1 | 0.0005 | 0 | 0.0000 |  |
|  | Kineosporiaceae | family | 1 | 0.0005 | 0 | 0.0000 |  |
|  | unclassified_Kineosporiaceae |  | 1 | 0.0005 | 0 | 0.0000 |  |
|  | Streptomycineae | suborder | 1 | 0.0005 | 0 | 0.0000 |  |
|  | Streptomycetaceae | family | 1 | 0.0005 | 0 | 0.0000 |  |
|  | Streptomyces | genus | 1 | 0.0005 | 0 | 0.0000 |  |
|  | unclassified_Propionibacterineae |  | 1 | 0.0005 | 265 | 0.1147 |  |
|  | unclassified_Actinomycetales |  | 413 | 0.1952 | 2344 | 1.0142 |  |
|  | Bifidobacteriales | order | 1 | 0.0005 | 0 | 0.0000 |  |
|  | Bifidobacteriaceae | family | 1 | 0.0005 | 0 | 0.0000 |  |
|  | Bifidobacterium | genus | 1 | 0.0005 | 0 | 0.0000 |  |
|  | Nitriliruptoridae | subclass | 33 | 0.0156 | 118 | 0.0511 |  |
|  | Euzebyales | order | 32 | 0.0151 | 101 | 0.0437 |  |
|  | Euzebyaceae | family | 32 | 0.0151 | 101 | 0.0437 |  |
|  | Euzebya | genus | 32 | 0.0151 | 101 | 0.0437 |  |
|  | unclassified_Actinobacteridae |  | 15 | 0.0071 | 148 | 0.0640 |  |
|  | unclassified_Nitriliruptoridae |  | 1 | 0.0005 | 17 | 0.0074 |  |
|  | Coriobacteridae | subclass | 1 | 0.0005 | 1 | 0.0004 |  |
|  | Coriobacteriales | order | 1 | 0.0005 | 1 | 0.0004 |  |
|  | Coriobacterineae | suborder | 1 | 0.0005 | 1 | 0.0004 |  |
|  | Coriobacteriaceae | family | 1 | 0.0005 | 1 | 0.0004 |  |
|  | unclassified_Coriobacteriaceae |  | 0 | 0.0000 | 1 | 0.0004 |  |
|  | Collinsella | genus | 1 | 0.0005 | 0 | 0.0000 |  |
|  | unclassified_Actinobacteria |  | 199 | 0.0941 | 335 | 0.1449 |  |
|  | unclassified_"Proteobacteria" |  | 1371 | 0.6480 | 2762 | 1.1950 |  |
|  |  |  |  |  |  |  |  |
|  | **Tenericutes** | **phylum** | **126** | **0.0596** | **3** | **0.0013** |  |
|  | Mollicutes | class | 126 | 0.0596 | 3 | 0.0013 |  |
|  | Mycoplasmatales | order | 25 | 0.0118 | 3 | 0.0013 |  |
|  | Mycoplasmataceae | family | 25 | 0.0118 | 3 | 0.0013 |  |
|  | Mycoplasma | genus | 25 | 0.0118 | 3 | 0.0013 |  |
|  | Acholeplasmatales | order | 20 | 0.0095 | 0 | 0.0000 |  |
|  | Acholeplasmataceae | family | 20 | 0.0095 | 0 | 0.0000 |  |
|  | Acholeplasma | genus | 20 | 0.0095 | 0 | 0.0000 |  |
|  | unclassified_Mollicutes |  | 81 | 0.0383 | 0 | 0.0000 |  |
|  |  |  |  |  |  |  |  |
|  | **Synergistetes** | **phylum** | **238** | **0.1125** | **121** | **0.0524** |  |
|  | Synergistia | class | 238 | 0.1125 | 121 | 0.0524 |  |
|  | Synergistales | order | 238 | 0.1125 | 121 | 0.0524 |  |
|  | Synergistaceae | family | 238 | 0.1125 | 121 | 0.0524 |  |
|  | Thermovirga | genus | 49 | 0.0232 | 0 | 0.0000 |  |
|  | Cloacibacillus | genus | 3 | 0.0014 | 0 | 0.0000 |  |
|  | unclassified_Synergistaceae |  | 186 | 0.0879 | 121 | 0.0524 |  |
|  |  |  |  |  |  |  |  |
|  | **Fusobacteria** | **phylum** | **461** | **0.2179** | **724** | **0.3132** |  |
|  | Fusobacteria | class | 461 | 0.2179 | 724 | 0.3132 |  |
|  | Fusobacteriales | order | 461 | 0.2179 | 724 | 0.3132 |  |
|  | Fusobacteriaceae | family | 434 | 0.2051 | 698 | 0.3020 |  |
|  | Fusobacterium | genus | 402 | 0.1900 | 586 | 0.2535 |  |
|  | Clostridium XIX | genus | 0 | 0.0000 | 4 | 0.0017 |  |
|  | Cetobacterium | genus | 1 | 0.0005 | 0 | 0.0000 |  |
|  | unclassified_"Fusobacteriaceae" |  | 31 | 0.0147 | 108 | 0.0467 |  |
|  | Leptotrichiaceae | family | 21 | 0.0099 | 14 | 0.0061 |  |
|  | Streptobacillus | genus | 4 | 0.0019 | 0 | 0.0000 |  |
|  | Sneathia | genus | 2 | 0.0009 | 1 | 0.0004 |  |
|  | Leptotrichia | genus | 1 | 0.0005 | 0 | 0.0000 |  |
|  | unclassified_"Leptotrichiaceae" |  | 14 | 0.0066 | 13 | 0.0056 |  |
|  | unclassified_"Fusobacteriales" |  | 6 | 0.0028 | 12 | 0.0052 |  |
|  |  |  |  |  |  |  |  |
|  | **Chloroflexi** | **phylum** | **259** | **0.1224** | **1031** | **0.4461** |  |
|  | Anaerolineae | class | 258 | 0.1220 | 1031 | 0.4461 |  |
|  | Anaerolineales | order | 258 | 0.1220 | 1031 | 0.4461 |  |
|  | Anaerolineaceae | family | 258 | 0.1220 | 1031 | 0.4461 |  |
|  | Anaerolinea | genus | 0 | 0.0000 | 1 | 0.0004 |  |
|  | unclassified_Anaerolineaceae |  | 258 | 0.1220 | 1030 | 0.4456 |  |
|  | unclassified_"Chloroflexi" |  | 1 | 0.0005 | 0 | 0.0000 |  |
|  |  |  |  |  |  |  |  |
|  | **Elusimicrobia** | **phylum** | **2** | **0.0009** | **0** | **0.0000** |  |
|  | Elusimicrobia | class | 2 | 0.0009 | 0 | 0.0000 |  |
|  | Elusimicrobiales | order | 2 | 0.0009 | 0 | 0.0000 |  |
|  | Elusimicrobiaceae | family | 2 | 0.0009 | 0 | 0.0000 |  |
|  | Elusimicrobium | genus | 2 | 0.0009 | 0 | 0.0000 |  |
|  |  |  |  |  |  |  |  |
|  | **OD1** | **phylum** | **1** | **0.0005** | **0** | **0.0000** |  |
|  | OD1_genera_incertae_sedis | genus | 1 | 0.0005 | 0 | 0.0000 |  |
|  | unclassified_Bacteria |  | 95860 | 45.3112 | 28266 | 12.2297 |  |
|  |  |  |  |  |  |  |  |
|  | **Total** |  | **211559** |  | **231126** |  |  |
|  |  |  |  |  |  |  |  |
|  |  |  |  |  |  |  |  |
|  |  |  |  |  |  |  |  |
|  | **Taxon** | **Rank** | **RT-SSU rRNA sequencing** | | **16S rRNA gene PCR amplicons** | |  |
|  |  |  | **Read number** | **% of total** | **Read number** | **% of total** |  |
|  |  |  |  |  |  |  |  |
|  | **Archaea** | **domain** | **16** | **100** | **0** | **0** |  |
|  |  |  |  |  |  |  |  |
|  | **Euryarchaeota** | **phylum** | **5** | **31.25** | **0** | **0** |  |
|  | Thermoplasmata | class | 2 | 12.5 | 0 | 0 |  |
|  | Thermoplasmatales | order | 2 | 12.5 | 0 | 0 |  |
|  | unclassified_Thermoplasmatales |  | 2 | 12.5 | 0 | 0 |  |
|  | Methanobacteria | class | 2 | 12.5 | 0 | 0 |  |
|  | Methanobacteriales | order | 2 | 12.5 | 0 | 0 |  |
|  | Methanobacteriaceae | family | 2 | 12.5 | 0 | 0 |  |
|  | Methanobrevibacter | genus | 2 | 12.5 | 0 | 0 |  |
|  | unclassified_"Euryarchaeota" |  | 1 | 6.25 | 0 | 0 |  |
|  | unclassified_Archaea |  | 11 | 68.75 | 0 | 0 |  |
|  |  |  |  |  |  |  |  |
|  | **Total** |  | **16** |  | **0** | **0** |  |
|  |  |  |  |  |  |  |  |
