# Supplementary material for: Characterising the Canine Oral Microbiome by Direct Sequencing of Reverse-Transcribed rRNA Molecules
Source: PLoS One. 2016 Jun 8;11(6):e0157046. doi: 10.1371/journal.pone.0157046 (PMC4898712; doi:10.1371/journal.pone.0157046)
Supplement: S3 Table — Primer sets were designed for use in qPCR experiments in order to assess the change in ratio of 16S rRNA gene copies resulting from amplification of an initial artificial 5-member microbial community subjected to 10, 20 and 30 cycles of PCR with the general bacterial primer set 63f 5’-GCCTAACACATGCAAGTC-3' and 518r 5’-ATTACCGCGGCTGCTGG-3'. (DOCX) [file pone.0157046.s006.docx]

**S3 Table**. **Genus-specific 16S rRNA gene PCR primer sets.** Primer sets were designed for use in qPCR experiments in order to assess the change in ratio of 16S rRNA gene copies resulting from amplification of an initial artificial 5-member microbial community subjected to 10, 20 and 30 cycles of PCR with the general bacterial primer set V1-V3F 5’-GCCTAACACATGCAAGTC-3' and V1-V3R 5’-ATTACCGCGGCTGCTGG-3'.

| **Primer** | **Specificity (genus)** | **Sequence** |
| --- | --- | --- |
| E3 Forward | *Cardiobacterium* | 5'-GCAGCACGAGAAAGC-3' |
| E3 Reverse | *Cardiobacterium* | 5'-ATCAGCGCGAGGTCT-3' |
| E9 Forward | *Fusobacterium* | 5'-CTCTTAGACCGGGAC-3' |
| E9 Reverse | *Fusobacterium* | 5'-GGGACGCAAAGCTCT-3' |
| A9 Forward | *Actinomyces* | 5'-ACGGGATCTGATGGG-3' |
| A9 Reverse | *Actinomyces* | 5'-CCCACAACCACCATG-3' |
| C10 Forward | *Treponema* | 5'-CGGCAAGAGAGAAGCTT-3' |
| C10 Reverse | *Treponema* | 5'-CTCTAACAGATGCGGTC-3' |
| F10 Forward | *Desulfomicrobium* | 5'-CCGGGAATGAGTAGAGT-3' |
| F10 Reverse | *Desulfomicrobium* | 5'-CATCCTTTACCGACTCC-3' |
