# Supplementary material for: Characterising the Canine Oral Microbiome by Direct Sequencing of Reverse-Transcribed rRNA Molecules
Source: PLoS One. 2016 Jun 8;11(6):e0157046. doi: 10.1371/journal.pone.0157046 (PMC4898712; doi:10.1371/journal.pone.0157046)
Supplement: S4 Table — (DOCX) [file pone.0157046.s007.docx]

**S4 Table. Features and advantages of the BION-meta software**

| Feature | BION-meta |
| --- | --- |
| Operating systems | Linux, Unix, Mac OSX. |
| Installation/Source | Installs to local computer from source with one short command. |
| Reference databases | Automatically installs Greengenes, RDP and SILVA databases. |
|  | Workflow uses complete ‘recipes’ that are easy to understand and edit. |
| Types of data analysed | Works with PCR amplicon and RT-SSU rRNA data. |
|  | Slices and de-replicates reference sub-database alignments to match gene region targeted by amplicons, which prevents mis-classification of reads to non-target gene regions and improves speed of classification. |
| Sequence input format | Accepts .sff files, fastq and fasta sequence formats and quality encodings from Sanger, Solexa and Illumina 1.3, 1.5 and 1.8. |
| Sequence quality control | Flexible parameters for de-multiplexing, cleaning, de-replication and clustering. |
| Similarity checker | Similarity matcher detects sequence reads with very low similarity and can ignore low quality regions of a sequence without throwing the read away, increasing the number of reads assigned. |
| Chimera detection | Includes a new fast chimera detection algorithm. Please refer to Supplementary Methods 1 for more information. |
| Taxonomic mapping | Ability to perform taxonomic mapping to species and sequence using multiple methods. |
| Combining sample datasets | Can merge and re-order taxonomic profiles. |
| Performance and usage | A taxonomic overview of 250k 454 reads takes 30-90 minutes on a four-core machine.  Run-time for 200 million Illumina reads is 2-6 hours (depending upon redundancy).  Maximum RAM usage is 1-2.5 Gb |
| Data output | Data outputs are compiled in a single flat directory with consistently named files. This directory is a static website containing all data analysis detailed statistics and can be copied, shared or published to the internet as is. Results include a copy of the source code used to generate the data. |
